# Screening and Preparation of Cocrystals: A Comparative Study of Mechanochemistry vs Slurry Methods

**DOI:** 10.1021/acs.cgd.1c00418

**Published:** 2021-06-09

**Authors:** Molly M. Haskins, Michael J. Zaworotko

**Affiliations:** Department of Chemical Sciences, Bernal Institute, University of Limerick, Limerick V94 T9PX, Ireland

## Abstract

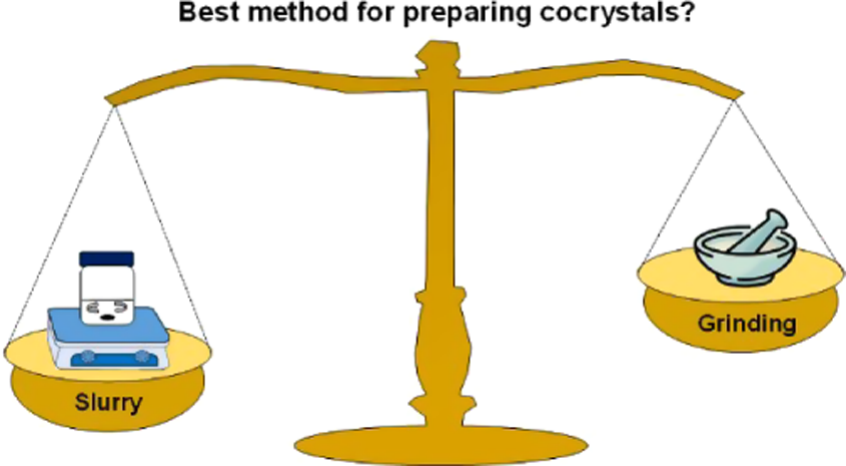

Cocrystals of biologically active molecular compounds have potential utility in drug products thanks to their effect upon physicochemical properties such as aqueous solubility. The fact that control of cocrystallization can be more challenging than crystallization of single-component crystals means that systematic studies that address the methodology of cocrystal screening, production, and purification are a topical subject. We previously reported a comparison of slow evaporation vs mechanochemistry for a library of 25 molecular cocrystals. Herein, we compare the previously reported mechanochemistry results (solvent-drop grinding (SDG) with eight solvents) with new results obtained from slurrying in five preferred solvents using the same library of 25 cocrystals. Overall, both methods were found to be effective with slurrying and SDG being 94 and 78.5% successful, respectively. Importantly, 96% of the cocrystals formed via slurrying were observed to be free of starting materials (coformers) according to powder X-ray diffraction (PXRD), whereas this was the case for only 72% of the cocrystals prepared by SDG. Slurrying therefore compared favorably with mechanochemistry, which tends to leave small amounts of unreacted coformer(s) as byproducts, and solution crystallization, which often affords crystals of the least soluble coformer because it can be difficult to control the saturation of three or more solids. Perhaps the most interesting and surprising result of this study was that water slurrying proved to be highly effective, even for low-solubility coformers. Indeed, water slurrying was found to be effective for 21 of the 25 cocrystals studied.

## Abstract 2

A comparative study of mechanochemistry and slurry methods for preparing a library of known cocrystals has revealed that slurrying, including water slurrying, offers favorable outcomes with respect to the yield and purity of cocrystal products.

## Introduction

Cocrystals have been defined as “solid single-phase crystalline materials made up of two or more different ionic and/or molecular components, generally in a stoichiometric ratio that are neither solvates, nor simple salts”.^1^ Crystal engineering of cocrystals has grown as a research subject over the last two decades thanks in part to the inherent amenability of most biological molecules to form pharmaceutical cocrystals through hydrogen-bonded interactions^2−8^ and the tendency of the resulting cocrystals to alter the physicochemical properties of a molecular compound without affecting its molecular structure.^6,8^ Cocrystals have thereby become relevant to the pharmaceutical industry as they can enhance the bioavailability of low-solubility molecular compounds,^9^ sometimes dramatically.^10^ A pharmaceutical cocrystal is composed of an active pharmaceutical ingredient (API) and at least one pharmaceutically acceptable coformer.^2^ To the best of our knowledge, at least eight pharmaceutical cocrystals have thus far been approved by regulatory bodies and marketed as drug products.^11^ In general, the potential utility of pharmaceutical cocrystals tends to be related to the Biopharmaceutics Classification System (BCS)^12^ class of the API in question. Specifically, Class II and IV APIs, those drug substances that exhibit low aqueous solubility, are particularly suitable candidates for cocrystallization studies. The utility of cocrystals is not limited to pharmaceuticals. They have also been studied in the context of nonlinear optical materials,^13^ molecular semiconductors,^14^ as a medium for stereocontrolled synthesis,^15^ and to enhance the performance of energetic materials (explosives, propellants, and pyrotechnics).^16^

**Scheme 1:**
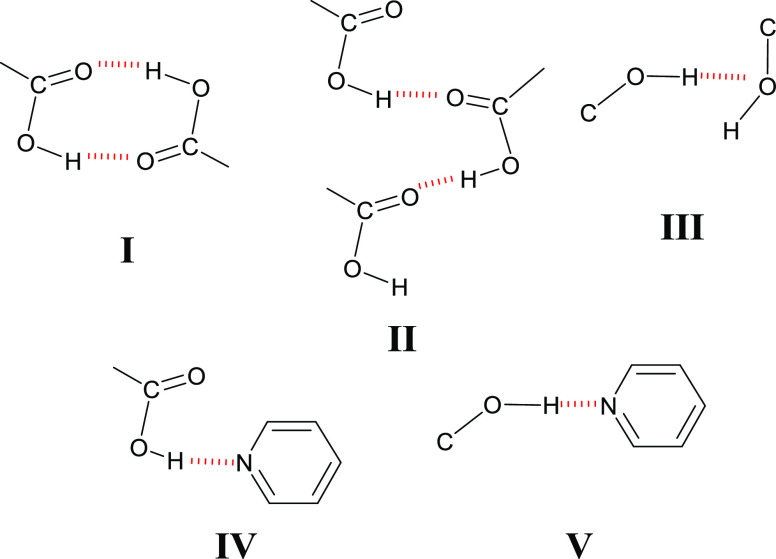
Supramolecular Homosynthons (I, II, and III) and Supramolecular Heterosynthons (IV and V) That Are Present in the Cocrystals of This Study

Interest in cocrystals results in part from their amenability to crystal engineering studies through the exploitation of supramolecular synthons, typically based upon complementary hydrogen bonding. The concept of supramolecular synthons was introduced by Desiraju in 1995,^17^ and the relevance of supramolecular heterosynthons, supramolecular synthons between different but complementary functional groups,^2^ to pharmaceutical cocrystal design using crystal engineering was recognized by several groups in 2003^5−7^ and 2004.^8^ Several studies have revealed that the hierarchy of supramolecular synthons is key to the design of cocrystals from first principles.^18−21^ Most relevant to the study herein are cocrystals containing phenol or carboxylic acid coformers and their tendency to form supramolecular heterosynthons with pyridyl moieties through OH···N_aromatic_ and COOH···N_aromatic_ interactions, respectively^22,23^ (Scheme 1, IV and V). Importantly, carboxylic acid moieties, phenolic groups, and pyridyl rings are widely encountered in APIs^7,24,25^ and FDA-approved coformers.^21^ These studies collectively emphasize the robust nature of these supramolecular heterosynthons and is a reason why we selected cocrystals based upon COOH···N_aromatic_ and OH···N_aromatic_ interactions for study.

Crystal engineering approaches to the design of certain families of cocrystals have reached a level of maturity thanks, at least in part, to the understanding gained from the hierarchy studies discussed above. This is not generally the case, however, for the methods used to discover and prepare cocrystals, where systematic studies that compare various approaches are quite few.^26^ With respect to discovery (screening) and preparation of cocrystals, mechanochemical grinding, solution evaporation, and slurrying are the most widely reported methods as discussed below.

Solid-state methods are used at the industrial scale, mainly with respect to inorganic solids and materials^27,28^ or to produce amorphous phases.^29^ Mechanochemistry refers to inducing reactivity in the solid state via input of mechanical energy. Its efficiency results from typically fast reaction times, generally high yields of product and little waste since only minimal amounts of solvent are needed during the grinding process.^30,31^ In recent years, mechanochemistry has evolved to include continuous processes such as twin-screw extrusion (TSE) (grinding of samples between two countering-rotating screwing elements)^32^ and hot-melt extrusion^33^ (melting of reactants). Supercritical fluid methods^34^ using supercritical CO_2_ as a solvent^35^ or antisolvent^36^ have also been studied and can eliminate the need for liquid solvent(s) altogether. With respect to the discovery and preparation of cocrystals, which typically occur at the milligram scale using either a pestle and mortar or ball mill, grinding has become a favored method.^37,38^ Multiple terms have been coined for this approach, including solvent-drop grinding^39,40^ (SDG), liquid-assisting grinding (LAG),^37^ or kneading.^41,42^ We adopt the term SDG herein. We note that, although SDG has wide acceptance with respect to cocrystal screening and preparation, grinding can result in crystalline defects that might generate partial amorphous content or incomplete conversion. In such situations, additional purification step(s) would be required.^43^

A slurry is a mixture of solid particles suspended in a liquid. Slurries can be used for bulk transportation of solids such as soil,^44^ but they are also used in a range of disciplines^45,46^ including for cocrystals.^47^ Using slurries is relatively non-labor-intensive and its variables can be optimized to afford high-purity products. The slurry technique also has advantages in the context of pharmaceutical materials since it typically results in thermodynamically stable products. For example, slurrying has been used to prepare the most stable cocrystal form^48^ under a given set of conditions and for the identification of thermodynamically stable polymorphs.^49^ Slurrying has been utilized for cocrystal screening^47,50^ and for scale-up of cocrystals for dissolution or solubility studies.^6,51^ Slurrying can also be used for solution-mediated phase transformations;^52^ Zhang’s group used slurries to prepare caffeine cocrystals,^53^ including one between caffeine and adipic acid,^54^ which could not be isolated by SDG according to a study from the Jones group.^55^ Zhang’s group also reported that cocrystal formation can be related to hydrate and solvate formation by molecular compounds based upon a thermodynamic understanding of their physical stability.^52^ We take a similar approach herein by selecting the parameters for our slurry experiments using solubility data from the pure coformers. Despite the larger consumption of solvent and longer reaction times compared to SDG, the use of greener or more “economically friendly” solvents such as water can overcome environmental concerns and some of the costs associated with solvent waste as discussed below.

Solution crystallization continues to be widely used to discover and prepare cocrystals.^18−21^ This technique involves controlled nucleation and growth of a cocrystal from a solution of its coformers under supersaturated conditions, typically induced by slow evaporation of solvent. The isolation of single crystals means that structural characterization by single-crystal X-ray diffraction (SCXRD) is a desirable outcome, although powder X-ray diffraction (PXRD) has been shown to be effective for the determination of cocrystal structures.^56^ Nevertheless, isolation of cocrystals from solution can be more challenging than for single-component crystals. Ternary phase diagrams can be generated to determine the conditions, typically a narrow range of conditions, under which cocrystals will be favored over low-solubility coformers.^26,57^ The relative amount of solvent and the relative solubility of the coformers in the specified solvent can be a decisive factor.^58^ For larger-scale production, slow evaporation can be time-consuming and produce large amounts of solvent waste. In general, the use of solvents is negative in terms of environmental impact but can be essential for the specifications needed for industrial production, e.g., for formulation and purification.^59^ A high rate of solvent consumption can lead to costly waste management and regimented safety procedures due to increased health risks. This poses the question of “what is a green solvent?” In 2007, Fischer^60^ attempted to address this question from two aspects: (i) environmental, health, and safety and (ii) energy demand. Slater and Savelski^61^ considered 12 environmental factors including the occupational hazard of a worker and their environment. Initiatives have been implemented since the mid-20th century to avoid the use of carcinogenic or ozone-depleting solvents, including the replacement of benzene with toluene in 1971^62,63^ and the ban of carbon tetrachloride in 1989.^64^

**Scheme 2:**
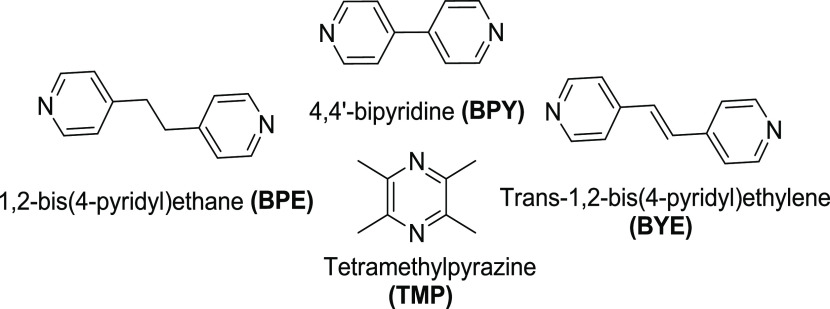
Pyridyl Coformers Present in Cocrystals 1–18

**Scheme 3:**
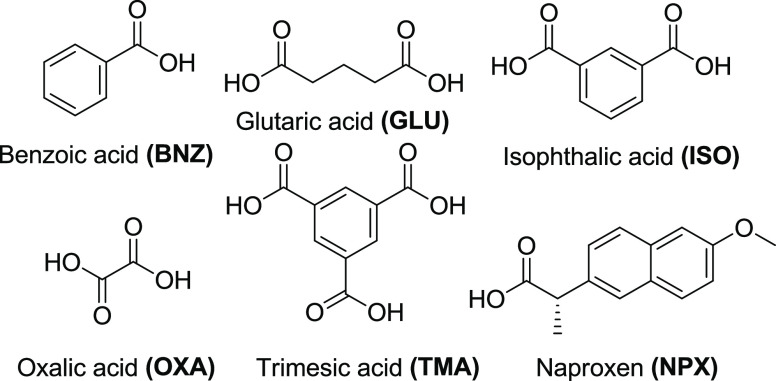
Carboxylic Acid Coformers Present in Cocrystals 1–9

**Scheme 4:**
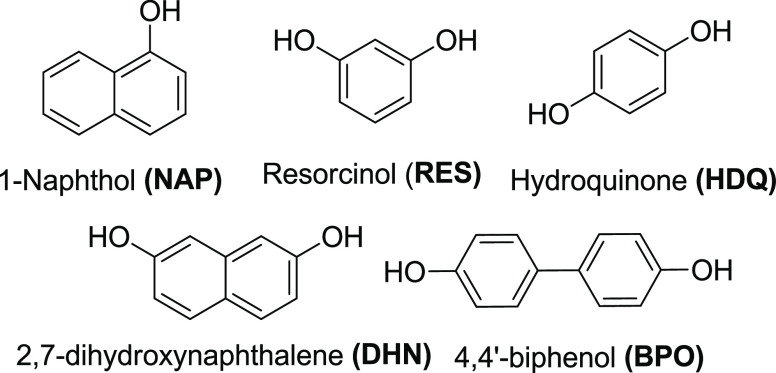
Phenolic Coformers Present in Cocrystals 10–17

**Scheme 5:**
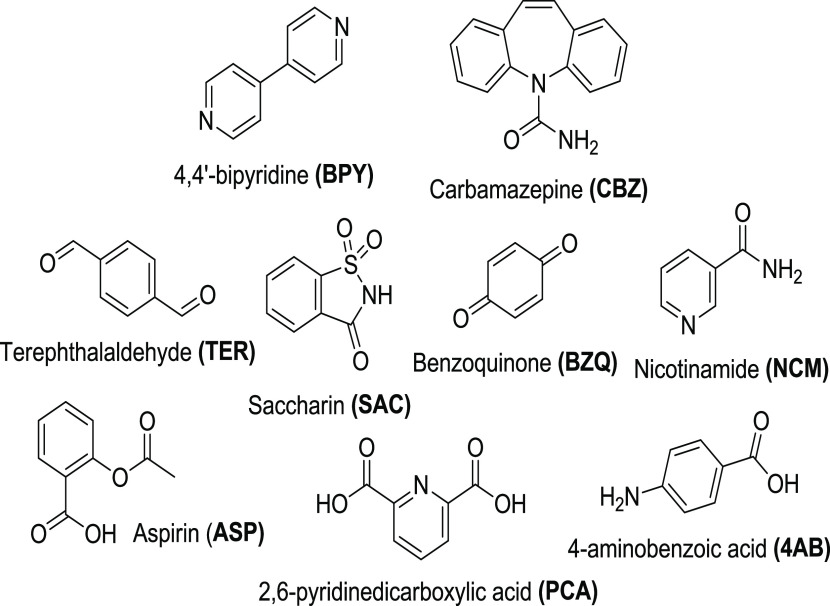
CBZ and Coformers in Cocrystals 18–25

Herein, we systematically address the utility of slurrying vs mechanochemistry for cocrystal preparation as a continuation of our previous study that compared solution crystallization and mechanochemistry with a library of 25 known cocrystals (Schemes 2–5).^65^ In addition to model compound coformers, carbamazepine (CBZ) was included in our previous study as CBZ is an example of a BCS Class II^66^ drug substance. CBZ is the API in the anticonvulsant drug product Tegretol.^67^ Herein, we use the same library of 25 cocrystals and therefore rely upon the SDG data reported previously. The solvents selected for our slurry experiments were water, methanol (MeOH), acetonitrile (MeCN), ethyl acetate (EtOAc), and 2-butanone, also commonly known as methyl ethyl ketone (MEK). These solvents offer different functionalities and different levels of polarity, i.e., aqueous/organic, alcohol/ketone, and protic/aprotic. Nonpolar and halogenated solvents were not considered for this study due to their increased health risks. This solvent choice was also guided by the green solvent selection guide for chemists by Clark et al.^68^ This guide noted that GSK and Pfizer have categorized MeCN as useable but problematic. However, it is one of Sanofi’s recommended solvents due to low cost and its common use in organic synthesis and purification, was therefore chosen for this study.

## Experimental Section

Reagents and solvents were obtained from Sigma-Aldrich (glutaric acid, isophthalic acid, trimesic acid, hydroquinone, benzoquinone, 4-aminobenzoic acid, oxalic acid, 1-naphthol, terephthaldehyde, saccharin, nicotinamide, resorcinol, and 2,6-pyridinecarboxylic acid) and TCI (benzoic acid, trans-1,2-bis-(4-pyridyl)ethylene, 4,4′-bipyridine, 1,2-bis-(4-pyridyl)ethane, 4,4′-biphenol, tetramethylpyrazine, naproxen, carbamazepine, aspirin, and 2,7-dihydroxynaphthalene) and used as received. X-ray powder diffraction (PXRD) was used for phase identification of coformers and cocrystals. We note that peak positions in experimental PXRD patterns can exhibit slight shifting compared to calculated PXRD patterns obtained from SCXRD data because of thermal expansion if data were collected at different temperatures.

### Powder X-ray Diffraction (PXRD)

PXRD studies of microcrystalline samples were performed in Bragg–Brentano geometry on a Panalytical Empyrean diffractometer (40 kV, 40 mA, Cu Kα_1,2_ (λ = 1.5418 Å)). A scan speed of 0.5 s/step (6°/min) with a step size of 0.05° in 2θ was used at ambient temperature.

### Solubility Test Using Gravimetric Method

A slurry containing each coformer was stirred for 24 h in 2 mL of deionized water, MeOH, MeCN, EtOAc, or MEK. The resulting slurry was filtered using Whatmann 0.45 μm poly(tetrafluoroethylene) (PTFE) syringe filters into a preweighed vial. The vial was reweighed and left in an oven at 40 °C until evaporation was complete. The vial was reweighed after drying.

### Slurry Experiments

Each slurry used 0.5 mL of solvent in a 10.5 mL vial with a diameter of 16 mm. A 10 mm stirring bar was placed into each vial and set to stir at 150 rpm for 24–48 h. The slurry was then filtered, washed with the solvent used for that slurry, and air-dried before being analyzed by PXRD. The relative amounts of coformer used for each slurry were based upon the stoichiometric ratio of the target cocrystal. The total amount of coformers used for each slurry was based on the solubility of each coformer to ensure that both coformers would be saturated under the conditions of the slurrying experiment. If the slurry experiment was subsequently deemed to be unsuccessful at ambient temperature, it was repeated at 30 °C. More details about the slurry experiments are presented in the Supporting Information.

### Mechanochemical Methods (as Previously Reported)

Coformers were subjected to grinding with an agate mortar and pestle for 4 min and thereafter characterized by infrared (IR) and PXRD experiments. In the event that partial conversion was achieved, a SPEX 8000 M Mixer/Mill was used for two stages of 10 min with the addition of a solvent prior to each stage. The stoichiometric ratios that the cocrystals exhibited from solution crystallization were used for grinding unless otherwise specified. Solvents used per 100 mg of cocrystal formers during SDG were as follows: MeOH 20 μL; EtOAc 20 μL; dimethylsulfoxide (DMSO) 4 μL; water 5 μL; toluene (Tol) 20 μL; cyclohexane (Cyclohex) 20 μL; chloroform 20 μL; dimethylformamide (DMF) 4 μL.

## Results and Discussion

### Design of Slurry Experiments

There are multiple variables associated with a slurry experiment that involves coformers. Two approaches were taken herein. Initially, an excess of the more soluble coformer used in each slurry was used, but this tended to result in isolation of the less soluble coformer as determined by PXRD. These preliminary results prompted us to take a different approach in which both coformers were saturated during the slurry experiment. Saturation was ensured by using the measured solubility of the more soluble coformer (Table 1) and adding an additional 10 mg of coformer. The appropriate stoichiometric amount of the second coformer was then determined and used. In essence, this approach follows that proposed by Zhang and co-workers^52^ by promoting nucleation of the cocrystal since; when both coformers are supersaturated, it is more likely that the system is in the appropriate region of ternary phase diagram to favor cocrystallization. This approach may be necessary for coformers that have a more stable hydrated phase compared to their cocrystal(s), e.g., CBZ dihydrate in the context of the CBZ cocrystals. The protocol might be further modified for scale-up, but for the purpose of the study herein, this approach was used without further modification.

**Table 1:** Solubility Data for Each Coformer in Water, MeOH, MeCN, EtOAc, and MEK

**compound name**	**water** (mg/mL)	**MeOH** (mg/mL)	**MeCN** (mg/mL)	**EtOAc** (mg/mL)
benzoic acid	<1.0	306.4	81.5	175.0
trans-1,2-bis(4-pyridyl)ethylene	<1.0	289.0	<1.0	<1.0
4,4′-bipyridine	1.8	456.7	84.1	134.6
glutaric acid	570.0	458.0	99.5	602.2
1,2-bis(4-pyridyl)ethane	<1.0	426.6	115.7	67.1
4,4′-biphenol	<1.0	57.1	13.3	56.1
tetramethylpyrazine	<1.0	<1.0	<1.0	<1.0
isophthalic acid	<1.0	10.8	2.0	3.4
trimesic acid	<1.0	<1.0	<1.0	1.8
hydroquinone	72.9	269.6	100.8	165.1
naproxen	<1.0	53.6	36.7	50.5
benzoquinone	1.8	4.8	15.2	57.7
carbamazepine	<1.0	63.4	43.5	12.7
4-aminobenzoic acid	2.7	146.9	57.9	75.5
oxalic acid	85.3	190.3	110.6	96.8
aspirin	2.0	221.8	63.3	25.4
1-naphthol	2.7	197.0	71.3	61.2
terephthalaldehyde	<1.0	221.5	76.2	79.7
saccharin	2.9	36.4	28.2	30.2
nicotinamide	419.1	180.2	21.2	10.8
2,7-dihydroxynaphthalene	4.5	379	76.2	79.7
resorcinol	566.5	544.4	525.8	443.5
2,6-pyridinecarboxylic acid	4.0	22.9	<1.0	<1.0

### Solubility Data

Solubility data was obtained for each coformer as presented in Table 1 and reveal that there is a broad range of solubility, both absolutely and relatively. For example, nicotinamide, glutaric acid, and resorcinol exhibit high aqueous solubilities (419.1, 570.0, and 566.5 mg/mL respectively), whereas 4, 4′-bipyridine and derivatives have an aqueous solubility of 1.6 mg/mL or less (cocrystals 5 and 6). Notably, this is representative of many pharmaceutical cocrystals, which are typically composed of an API with a low aqueous solubility and a high-solubility coformer.^6,8^

#### Cocrystals Containing the COOH···N_aromatic_ Supramolecular Heterosynthon (1–9)

The results obtained from carboxylic acid and pyridyl coformers are presented in Table 2 and are color-coded according to the outcome. As revealed by Table 2, cocrystals 1–8 were formed via SDG and slurry for all solvents used as determined by a comparison of experimental and calculated PXRD data (Figures S1–S8). However, in most SDG experiments, it was observed that, although the experimental PXRD pattern matched that of the calculated PXRD pattern of the target cocrystal, there were additional peaks corresponding to pure coformers. In such situations, additional milling was conducted to promote complete conversion from coformers to cocrystal. With respect to SDG, inefficient mixing caused by clumping of particles of the coformers could be the reason for incomplete conversion to cocrystal. Cocrystal 9, the cocrystal of trimesic acid and trans-1,2-bis(4-pyridyl)ethylene, only formed in DMSO and DMF through SDG. An unknown phase resulted from SDG using toluene, whereas physical mixtures of coformers were isolated from SDG involving water, MeOH, or EtOAc. Interestingly, the slurrying experiments afforded cocrystal 9 with all five solvents used, three of which were solvents that were used unsuccessfully for the SDG experiments (Figure S9). We note that even coformers with an aqueous solubility of <1 mg/mL formed via slurry in water, e.g., cocrystal 4. Such a situation is favorable, as one would expect a high overall yield as relatively low quantities of coformers remain in solution (assuming the cocrystal does not exhibit a substantial increase in solubility compared to the parent coformers). Overall, the results presented in Table 2 suggest that slurrying might be better suited to produce high-purity products than SDG.

**Table 2:**
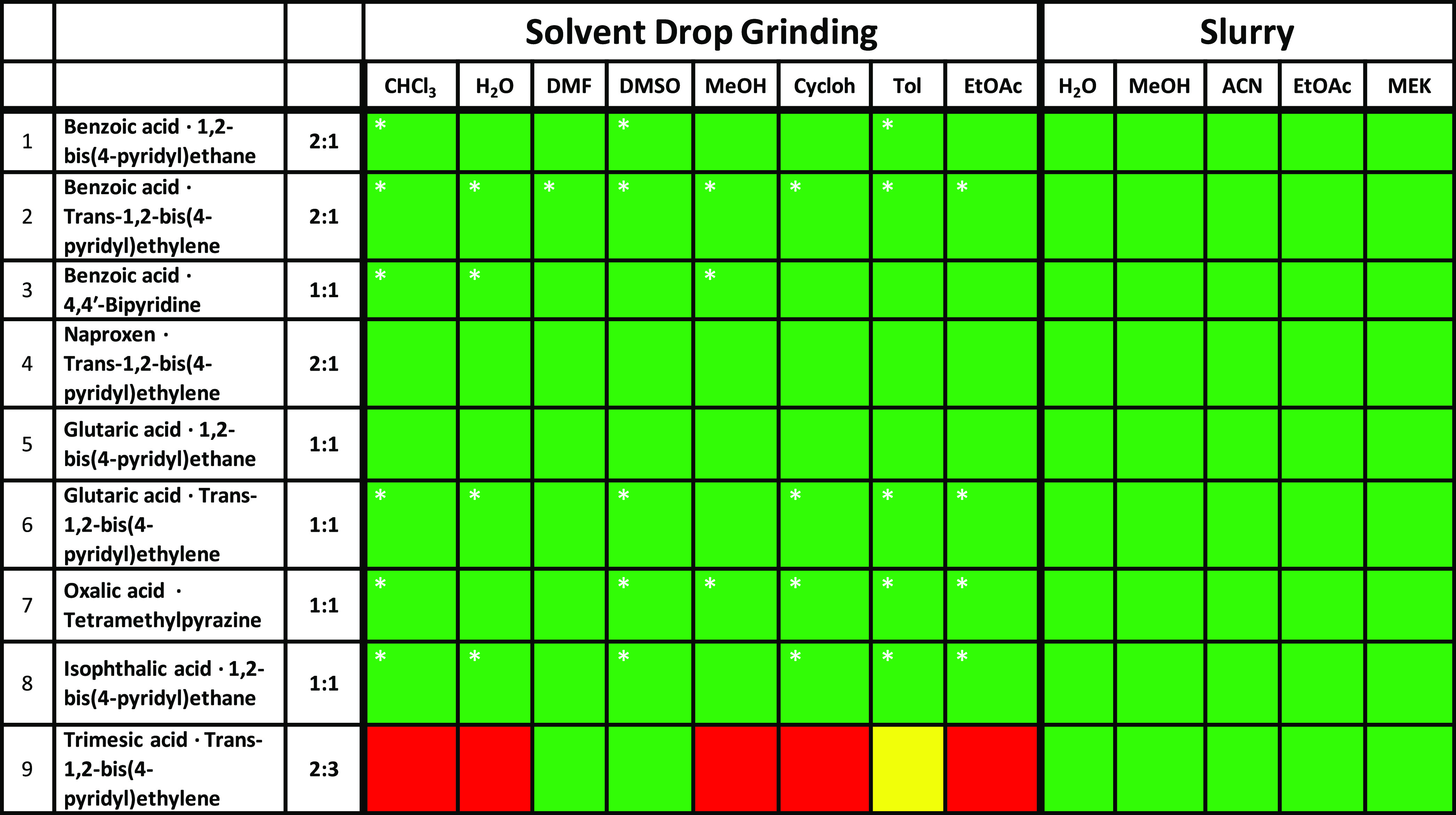
Comparison of the Results of Slurry and SDG^65^ Experiments for Cocrystals 1–9^a^

a Red = physical mixture of pure coformers, green = cocrystal formed, yellow = unidentified form. *Indicates the presence of pure coformers as measured by PXRD (see the SI for details).

#### Cocrystals Containing the OH···Narom Supramolecular Heterosynthon (10–17)

The results obtained from phenol and pyridyl coformers are presented in Table 3 and are color-coded according to the outcome. Cocrystals 10–13 were isolated from all solvents tested via SDG and slurry (Figures S10–S12). However, 10 of the SDG experiments (40% of the experiments conducted) contained additional PXRD peaks corresponding to pure coformers as following analysis of calculated PXRD patterns. Conversely, only 5% of the slurry experiments exhibited additional PXRD peaks. Cocrystal 13, the 1:1 cocrystal of 4,4′-phenol and trans-1,2-bis(4-pyridyl)ethylene, exhibited an additional peak in its water slurry PXRD pattern at approximately 23°, which correlates to a peak from 4,4′-biphenol (Figure S13). Cocrystal 14 formed in all slurry solvents (Figure S14). With respect to the SDG method, cocrystal 14 formed in the same solvents used for slurry (H_2_O, MeOH, and EtOAc), DMF, and DMSO. Cocrystal 14 did not form, however, from SDG in CHCl_3_, Tol, or Cycloh. Interestingly, in attempts to prepare cocrystal 15 of hydroquinone and tetramethylpyrazine via SDG, the three solvents that did not produce 14 produced an unknown phase with PXRD peaks that neither matched the targeted cocrystals nor the pure coformers. This phase has not been structurally characterized and is likely to be a polymorph (of the cocrystal or of the pure coformers), a different stoichiometry cocrystal or a solvate/hydrate of a cocrystal. Cocrystal 15 was afforded from the remaining SDG solvents, and all of the slurry solvents although PXRD peaks corresponding to coformer were observed when water was used as a solvent for SDG (Figure S15). With the exception of water for the slurry method, cocrystal 16 formed in all solvents used for both SDG and slurry. An unknown phase was obtained from the water slurry of resorcinol and tetramethylpyrazine, its PXRD pattern being different from polymorphs or hydrates of the cocrystal or the individual coformers archived in the Cambridge Structural Database (CSD; version 5.42, February 2021) (Figure S16). An issue with the slurry experiments of cocrystal 15 was that the solubility of resorcinol in all selected solvents is greater than 500 mg/mL, therefore requiring a large amount of coformer and additional solvent to achieve efficient mixing. This would be problematic for scale-up using slurry thus SDG would likely be preferred over slurry for this cocrystal. Cocrystal 17 neither formed by SDG nor slurry in water, instead resulting in an unknown phase similar to cocrystal 16. Once again, the PXRD pattern of the solids isolated did not match any of the relevant solid forms archived in the CSD. Nevertheless, 17 was successfully isolated in the other selected solvents (Figure S17).

**Table 3:**
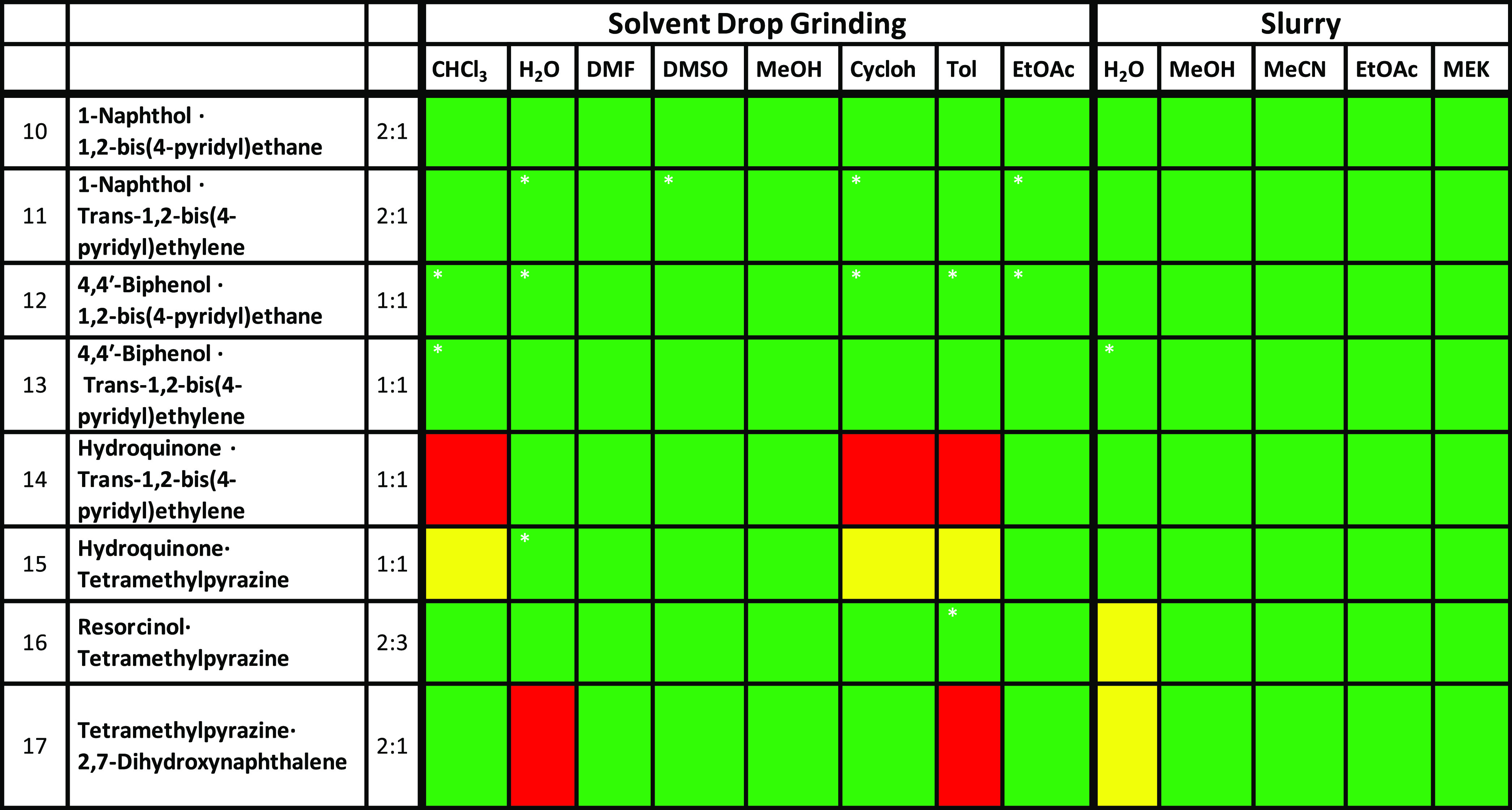
Comparison of the Results of Slurry and SDG^65^ Experiments for Cocrystals 10–17^a^

a Red = physical mixture of pure coformers, green = cocrystal formed, yellow = unidentified form, *Indicates the presence of pure coformers as measured by PXRD (see the SI for details).

#### Cocrystals Containing Carbamazepine, CBZ (18–25)

CBZ is a BCS class II API that was one of the first APIs studied in the context of systematic cocrystallization studies.^69^ CBZ has been widely studied since, partly as a result of its promiscuity in terms of crystal forms; a CSD survey (version 5.42, February 2021) revealed that 148 structures containing CBZ are archived in the CSD, including more than 61 cocrystals, 17 solvates, and 5 polymorphs. The results obtained for CBZ cocrystals are presented in Table 4 and are color-coded according to the outcome. Cocrystal 18, a 2:1 cocrystal between CBZ and 4,4′-bipyridine, formed in all SDG experiments including that with water. When the slurry method was applied in water, however, the resulting PXRD pattern (Figure S18) indicates that dihydrate form of CBZ (REFCODE/FEFNOT01) had been isolated. A 1:1 stoichiometry was targeted for cocrystal 19 between 4-aminobenzoic acid (4AB)/CBZ. Rather, a 2:1 cocrystal was afforded, with water and EtOAc generating the hydrated form of the 2:1 cocrystal (Figure S19). Rodríguez-Hornedo’s and co-workers^70^ investigated the stability of these cocrystals and concluded that the 1:1 cocrystal is more stable at higher 4AB concentrations, whereas the 2:1 cocrystal is favored at lower 4AB concentrations. As the relative solubilities of CBZ and 4AB are similar in all slurry solvents used, their work supports our isolation of the 2:1 cocrystals. Conversely, the expected 1:1 stoichiometry cocrystal was obtained via SDG using MeOH and EtOAc, with the hydrated 1:1 cocrystal being isolated from water and DMSO. The remaining solvents afforded a physical mixture of the two coformers. Cocrystal 20 was afforded in all five slurry solvents but did not form when the same solvents were used in SDG with the exception of water (Figure S20). The 2:1 cocrystal of benzoquinone and CBZ, cocrystal 21, was afforded when coformers were ground in the presence of MeOH, DMSO, and DMF or slurried in water, MeOH, MeCN, EtOAc, or MEK (REFCODE/UNEYOB). Excess CBZ was identified in the PXRD pattern obtained after water slurry, whereas physical mixtures were produced in the remaining SDG experiments (Figure S21). Cocrystal 22 did not form by water slurry but was afforded via SDG in the presence of water. Cocrystal 22 resulted from the remaining slurry solvents, with CBZ present in the PXRD patterns of the solids isolated from MeOH, MeCN, and MEK (Figure S22). CBZ cocrystals 23 and 24, containing saccharin and nicotinamide, respectively, were isolated from slurry and SDG in the same solvents with the exception of water for cocrystal 23 (Figures S23 and S24). The 1:1 cocrystal between CBZ and aspirin only formed in DMSO using SDG but formed readily from slurrying in water, MeOH, EtOAc, and MEK. The anhydrous form of CBZ is the product of the MeCN slurry.

**Table 4:**
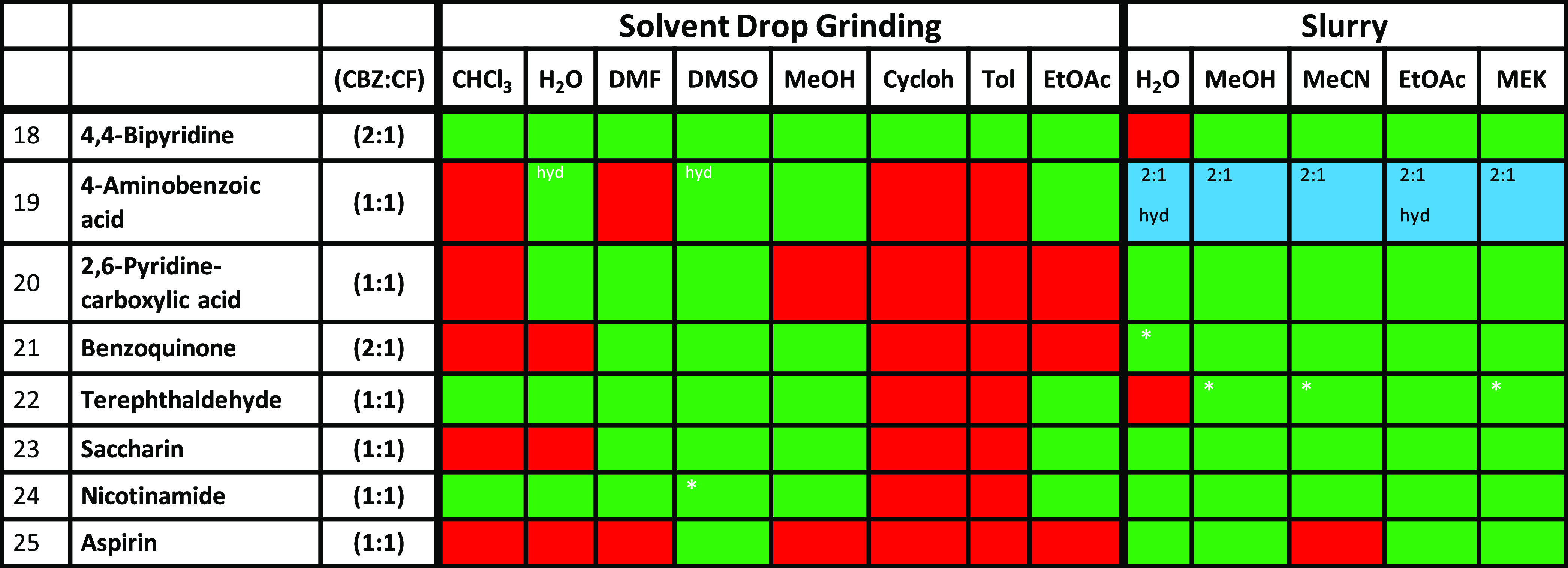
Comparison of the Results of Slurry and SDG^65^ Experiments for Carbamazepine (CBZ) Cocrystals 18–25^a^

a Red = physical mixture of pure coformers, green = cocrystal formed, blue = different-stoichiometry cocrystal than that targeted. *Indicates the presence of pure coformers as measured by PXRD (see the SI for details).

#### Analysis of Results

It is well recognized that cocrystals can afford drug substances with improved physicochemical properties, but it is unclear which methodology is most suitable for screening and production of cocrystals. The study herein compares the three most commonly used methodologies, slow evaporation, mechanochemistry, and slurry, with respect to their ability to produce cocrystals. In our earlier study, we compared slow evaporation from solution with mechanochemistry.^65^ We therein indicated that, although solution crystallization has the advantage of generating suitable crystals for SCXRD analysis, there are pitfalls. In particular, outcomes are heavily impacted by the relative solubility of the coformers and the ternary phase diagram can change dramatically from one solvent to another. The effect of solvent means that many attempts to optimize a solvent system may be required, making the process of determining the optimal crystallization conditions time-consuming. In essence, isolation of a specific cocrystal through solution crystallization is more challenging than isolation of single-component crystals because the regions of the ternary phase diagram that thermodynamically favor a specific cocrystal can be quite narrow.^26,57,58^ In addition, when scaling up solution crystallization processes, large amounts of solvent waste are expected to be produced.

Interest in the use of mechanochemical methods for the discovery and synthesis of materials has grown as they can offer fast reaction times, high yield, and use little or no solvent, meaning low waste and high atom economy.^30^ In terms of screening, mechanochemistry can take just minutes and is therefore faster than other methods. SDG can also be advantageous since it is largely unaffected by the relative solubility of the individual coformers. This is evident for cocrystals that are composed of highly soluble coformers. For example, resorcinol (cocrystal 23) has a solubility of >500 mg/mL in all of the slurry solvents and SDG proved to be the most suitable method for this particular cocrystal. Overall, we observed a 78.5% success rate for preparing cocrystals by SDG, although over a quarter (28%) of the solids produced physical mixtures of coformers and cocrystals. As a consequence, additional purification step(s) may be required, such as recrystallization. The degree of scale-up of cocrystals by mechanochemistry might also be problematic as ball milling is a batch process that is prone to clumping of solids and heterogeneous outcomes. The development of twin-screw extrusion (TSE) mills offers the possibility of a continuous process for synthesizing cocrystals and other classes of materials, but there are some drawbacks to TSE.^71^

The slurry method proved to be most successful as several cocrystals that were not afforded by SDG were formed via slurry in the same set of solvents. Further, the slurry method is facile, requires less labor, and the needed equipment is available in most laboratories. Herein, we observed a 94% success rate for slurry using the five preferred^68^ solvents selected for study. Interestingly, only 4% of the resulting cocrystals contained coformer as an impurity compared to 28% of the SDG experiments. The gathering of preliminary solubility data was crucial to select suitable conditions for our slurry experiments. Our study encompassed a broad range of solubilities, both absolutely and relatively, as exemplified by cocrystal 5, which formed in all five solvents. Cocrystal 5 is a 1:1 cocrystal between glutaric acid and 1,2-bis(4-pyridyl)ethane, and the coformers exhibit similar solubility in methanol (458.0 mg/mL an 426.6 mg/mL, respectively) but a difference of >500 mg/mL in water and EtOAc (Table 1). On the contrary, cocrystals for which both coformers exhibit a solubility of <1 mg/mL were cocrystal 1, benzoic acid, and trans-1,2-bis(4-pyridyl)ethylene in water.

It is perhaps an unexpected finding of this study that water proved to be an appropriate solvent for synthesizing cocrystals with an 80% success rate across slurry and SDG experiments. Water is not an ideal solvent for solution crystallization as APIs targeted for cocrystallization tend to exhibit low aqueous solubility and many cocrystals are metastable at the stoichiometry of the cocrystal.^70^ Nevertheless, from an industry perspective, water is probably the most desirable solvent for safety, economic, and environmental reasons.^68^ Further, if the goal of cocrystallization screening is to increase the aqueous solubility of an API, then water slurry could be optimal as when cocrystals are produced, it is likely to be in high yield. Even CBZ, which readily forms a dihydrate, formed cocrystals in water. Moreover, slurrying is likely to directly afford thermodynamically stable solid forms,^49^ a feature desirable in pharmaceutical dosage forms as it reduces the risks of phase changes occurring during the later stages of drug development (e.g., during manufacturing and storage).

This study herein does not address other factors that are relevant from a practical utility perspective, including crystallinity, particle size, and bulk purity, each of which can impact dissolution rate and tabletability.^72^ For example, Rahman and co-workers^73^ compared the dissolution rate of an acyclovir-succinic acid cocrystal made via either slurrying or grinding. They reported that the cocrystal formed by slurrying exhibited a faster dissolution rate compared to the poorly crystalline product afforded by grinding.

## Conclusions

In conclusion, solution crystallization is the traditional method for preparing cocrystals but can require many trial-and-error experiments because the solid form obtained is sensitive to the concentration and solvent used as illustrated by ternary phase diagrams.^26,57,58^ Ternary phase diagrams are influenced by the relative solubility of the pure coformers; coformers with similar solubility values are more likely to undergo congruent dissolution compared to coformers that are of very different solubility. This was highlighted by Peterson *et al.*, who reported very different ternary phase diagrams in water versus methanol for the 1:1 cocrystal of *trans*-cinnamic acid and nicotinamide.^57^ The study herein revealed that both SDG and slurry methodologies resulted in high success rates: slurry was successful in 94% of attempts; 78.5% of the SDG attempts were successful. Several cocrystals that did not form by SDG were readily afforded via slurry in the same solvent, or vice versa. We conclude from these results that supersaturation conditions played a key role in the overall success rate of both methodologies. We note that SDG and slurry as conducted herein result in saturation of both coformers. Therefore, our results are consistent with previous studies, which reported that increasing coformer concentration resulted in decreasing the solubility of the targeted cocrystal.^26,58^

In terms of comparing SDG with slurry, SDG is advantageous because it can produce cocrystals fast and in high yield with little or no solvent, but purity of products can be an issue (72% of cocrystals isolated by SDG were pure according to PXRD). Subsequent purification of cocrystals adds an additional step that can reduce the yield of a cocrystal or worse if supersaturation conditions are not maintained during washing (the least soluble coformer may precipitate).^52^ The slurry method, on the other hand, resulted in cocrystal products that were PXRD pure 96% of the time. On balance, based upon the library of cocrystals studied herein, we consider that slurrying, even water slurrying, offers a facile and effective approach for cocrystal discovery and production. Indeed, that water proved to be a suitable solvent for both slurrying and SDG is perhaps the most surprising and relevant aspect of the results we report herein.

## Supporting Information Available

Full details of each slurry experiment for the 25 cocrystals studied herein and PXRD patterns of products and starting materials (Figures S1–25) (PDF)
