# Supplementary material for: Screening and Preparation of Cocrystals: A Comparative Study of Mechanochemistry vs Slurry Methods
Source: Cryst Growth Des. 2021 Jun 9;21(7):4141–50. doi: 10.1021/acs.cgd.1c00418 (PMC8273892; doi:10.1021/acs.cgd.1c00418)
Supplement: Supplementary file 1 — cg1c00418_si_001.pdf [file cg1c00418_si_001.pdf]

Screening and Preparation of Cocrystals: A Comparative Study of Mechanochemistry vs. Slurry Methods.

Molly May Haskins[a] and Michael J. Zaworotko[a]*

[a]Department of Chemical Sciences, Bernal Institute, University of Limerick, Ireland

Supporting Information

Contents

Preliminary solubility test using gravimetric method. 3

Slurry experiments: 3

Slurry 1: Benzoic acid · 1,2-bis(4-pyridyl)ethane (2:1) [REFCODE: COZXIZ] 4

Slurry 2: Benzoic acid · trans-1,2-bis(4-pyridyl)ethylene (2:1) [REFCODE: COZXOF] 4

Slurry 3: Benzoic acid · 4,4′ -Bipyridine (1:1) [REFCODE: COZXUL] 5

Slurry 4: Naproxen · Trans-1,2-bis(4-pyridyl)ethylene (2:1) [REFCODE: COZYAS] 6

Slurry 5: Glutaric acid · 1,2-bis(4-pyridyl)ethane (1:1) [REFCODE: COZYEW] 7

Slurry 6: Glutaric acid · Trans-1,2-bis(4-pyridyl)ethylene (1:1) [REFCODE: COZYIA] 8

Slurry 7: Oxalic acid · Tetramethylpyrazine (1:1) [REFCODE: COZZOH] 9

Slurry 8: Isophthalic acid · 1,2-bis(4-pyridyl)ethane (1:1) [REFCODE: COZYUM] 10

Slurry 9: Trimesic acid · 1,2-Bis(4-pyridyl)ethane (1:2) [REFCODE: COZXEV] 11

Slurry 10: 1-Naphthol · 1,2-bis(4-pyridyl)ethane (2:1) [REFCODE: COZZAT] 12

Slurry 11: 1-Naphthol · trans-1,2-bis(4-pyridyl)ethylene (2:1) [REFCODE: QOVHOV] 13

Slurry 12: 4,4′ -Biphenol · 1,2-bis(4-pyridyl)ethane (1:1) [REFCODE: COZZEX] 14

Slurry 13: 4,4′ -Biphenol · Trans-1,2-bis(4-pyridyl)ethylene (1:1) [REFCODE: QOVHUF] 15

Slurry 14: Hydroquinone · Trans-1,2-bis(4-pyridyl)ethylene (1:1) [REFCODE: COZZIB] 16

Slurry 15: Hydroquinone · Tetramethylpyrazine (1:1) [REFCODE: COZZOH] 17

Slurry 16: Resorcinol · Tetramethylpyrazine (2:3) [REFCODE: COZZUN] 18

Slurry 17: 2,7-Dihydroxynaphthalene · Tetramethylpyrazine (1:2) [REFCODE: CUBBAD] 19

Slurry 18: 4, 4’-Bipyridine · Carbamazepine (1:2) [REFCODE: XAQQUC] 20

Slurry 19: 4-Aminobenzoic acid · Carbamazepine (1:1) [REFCODE: XOXHEY] 21

Slurry 20: 2, 6-Pyridinecarboxylic acid · Carbamazepine (1:1) [REFCODE: XAQRIR] 22

Slurry 21: Benzoquinone · Carbamazepine (1:2) [REFCODE: UNEYOB] 23

Slurry 22: Terephthalaldehyde · Carbamazepine (1:2) [REFCODE: UNEYUH] 24

Slurry 23: Saccharin · Carbamazepine (1:1) [REFCODE: UNEZAO] 25

Slurry 24: Nicotinamide · Carbamazepine (1:1) [REFCODE: UNEZES] 26

Slurry 25: Aspirin · Carbamazepine (1:1) [REFCODE: TAZROA] 27

Preliminary solubility test using gravimetric method.

A slurry containing each coformer was stirred for 24 hours in 2 mL of deionized water, MeOH, MeCN, EtOAc or MEK. The slurry was filtered using Whatmann 0.45 μm PTFE syringe filters into a pre-weighed vial. The vial was reweighed and left in an oven at 40°C until evaporation was complete. The vial was reweighed after drying. Solubility tests were conducted on the following compounds; benzoic acid, trans-1,2-bis(4-pyridyl)ethylene, 4,4'-bipyridine, glutaric acid, 1,2-bis(4-pyridyl)ethane, 4,4'-biphenol, tetramethylpyrazine, isophthalic acid, trimesic acid, hydroquinone, naproxen, benzoquinone, carbamazepine, 4-aminobenzoic acid, oxalic acid, aspirin, 1-naphthol, terephthalaldehyde, saccharin, nicotinamide, 2,7-dihydroxynaphthalene, resorcinol and 2,6-pyridinecarboxylic acid.

Slurry experiments:

Each slurry used 0.5 mL of solvent in a 10.5 mL vial with a diameter of 16 mm. A 10 mm stirring bar was placed into each vial and set to stir at 150 rpm for 24-48 h. The slurry was then filtered, washed with the solvent used for that slurry and air dried before being analyzed by PXRD. The relative amounts of coformer used for each slurry were based upon the stoichiometric ratio of the target cocrystal. The total amount of coformers used for each slurry was based on the solubility of each coformer to ensure that both coformers would be saturated during the slurrying experiment. If the slurry experiment was subsequently deemed to be unsuccessful at ambient temperature, it was repeated at 30°C.

Slurry 1: Benzoic acid · 1,2-bis(4-pyridyl)ethane (2:1) [REFCODE: COZXIZ]

Benzoic acid (100 mg, 0.82 mmol) and 1,2-bis(4-pyridyl)ethane (75 mg, 0.41 mmol) were added to 0.5 mL deionized water and left to stir for 48 hours at room temperature. Benzoic acid (291 mg, 2.39 mmol) and 1,2-bis(4-pyridyl)ethane (220 mg, 1.19 mmol) were added to 0.75 mL methanol and left to stir for 24 hours at room temperature. Benzoic acid (100 mg, 0.82 mmol) and 1,2-bis(4-pyridyl)ethane (75 mg, 0.41 mmol) were added to 0.5 mL acetonitrile and left to stir for 24 hours at room temperature. Benzoic acid (150 mg, 1.22 mmol) and 1,2-bis(4-pyridyl)ethane (112 mg, 0.62 mmol) were added to 0.5 mL ethyl acetate and left to stir for 24 hours at room temperature. Benzoic acid (200 mg, 1.64 mmol) and 1,2-bis(4-pyridyl)ethane (150 mg, 0.82 mmol) were added to 0.5 mL methyl ethyl ketone and left to stir for 24 hours at room temperature.

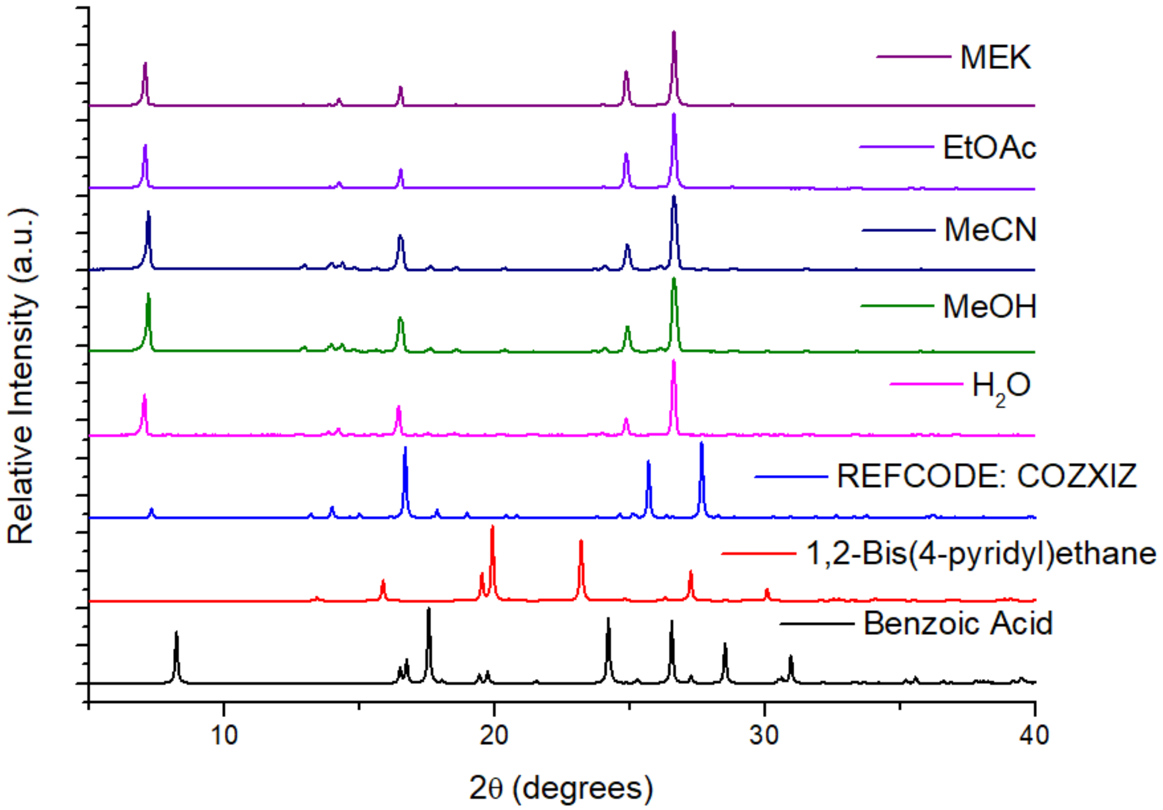

***Figure S1**. PXRD patterns from slurries in (top) MEK, EtOAc, MeCN, MeOH, water, cocrystal 1 calculated PXRD and the two starting materials.*

Slurry 2: Benzoic acid · trans-1,2-bis(4-pyridyl)ethylene (2:1) [REFCODE: COZXOF]

Benzoic acid (100 mg, 0.82 mmol) and trans-1,2-bis(4-pyridyl)ethylene (75 mg, 0.41 mmol) were added to 0.5 mL deionized water and left to stir for 48 hours at room temperature. Benzoic acid (180 mg, 1.6 mmol) and trans-1,2-bis(4-pyridyl)ethylene (145 mg, 0.8 mmol) were added to 0.5 mL MeOH and left to stir for 24 hours at room temperature. Benzoic acid (80 mg, 0.66 mmol) and trans-1,2-bis(4-pyridyl)ethylene (61 mg, 0.33 mmol) were added to 0.5 mL acetonitrile and left to

stir for 24 hours at room temperature. Benzoic acid (150 mg, 1.23 mmol) and trans-1,2-bis(4-pyridyl)ethylene (112 mg, 0.62 mmol) were added to 0.5 mL ethyl acetate and left to stir for 24 hours at room temperature. Benzoic acid (150 mg, 1.23 mmol) and trans-1,2-bis(4-pyridyl)ethylene (112 mg, 0.62 mmol) were added to 0.5 mL methyl ethyl ketone and left to stir for 24 hours at room temperature.

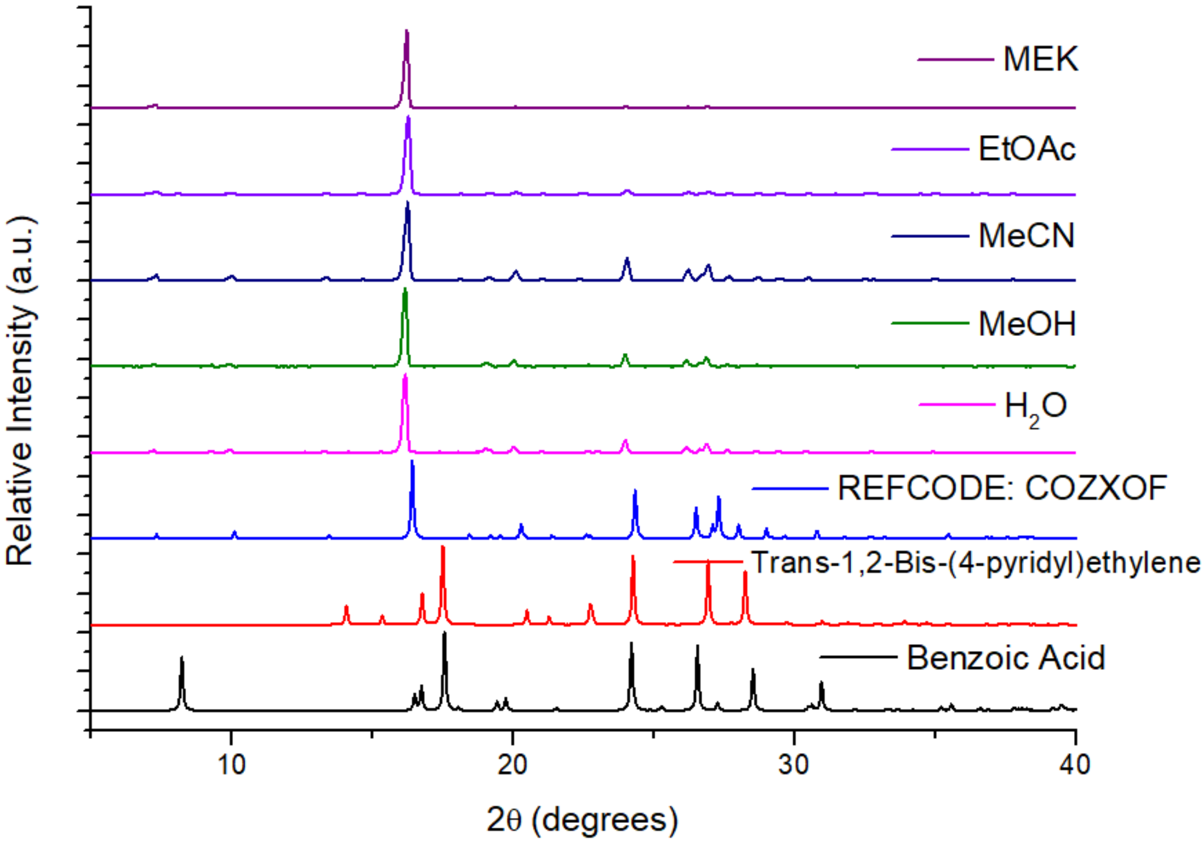

***Figure S2**. PXRD patterns from slurries in (top) MEK, EtOAc, MeCN, MeOH, water, cocrystal 2 calculated PXRD and the two starting materials.*

Slurry 3: Benzoic acid · 4,4′-Bipyridine (1:1) [REFCODE: COZXUL]

Benzoic acid (50 mg, 0.41 mmol) and 4,4′-bipyridine (64 mg, 0.41 mmol) were added to 0.5 mL deionized water and left to stir for 48 hours at 30°C. Benzoic acid (187 mg, 1.53 mmol) and 4,4′-bipyridine (240 mg, 1.53 mmol) were added to 0.75 mL methanol and left to stir for 24 hours at 30°C. Benzoic acid (108 mg, 0.88 mmol) and 4,4′-bipyridine (128 mg, 0.88 mmol) were added to 0.5 mL acetonitrile and left to stir for 24 hours at 30°C Benzoic acid (100 mg, 0.82 mmol) and 4,4′-bipyridine (128 mg, 0.82 mmol) were added to 0.5 mL ethyl acetate and left to stir for 24 hours at 30°C. Benzoic acid (150 mg, 1.23 mmol) and 4,4′-bipyridine (192 mg, 1.23 mmol) were added to 0.5 mL methyl ethyl ketone and left to stir for 24 hours at 30°C.

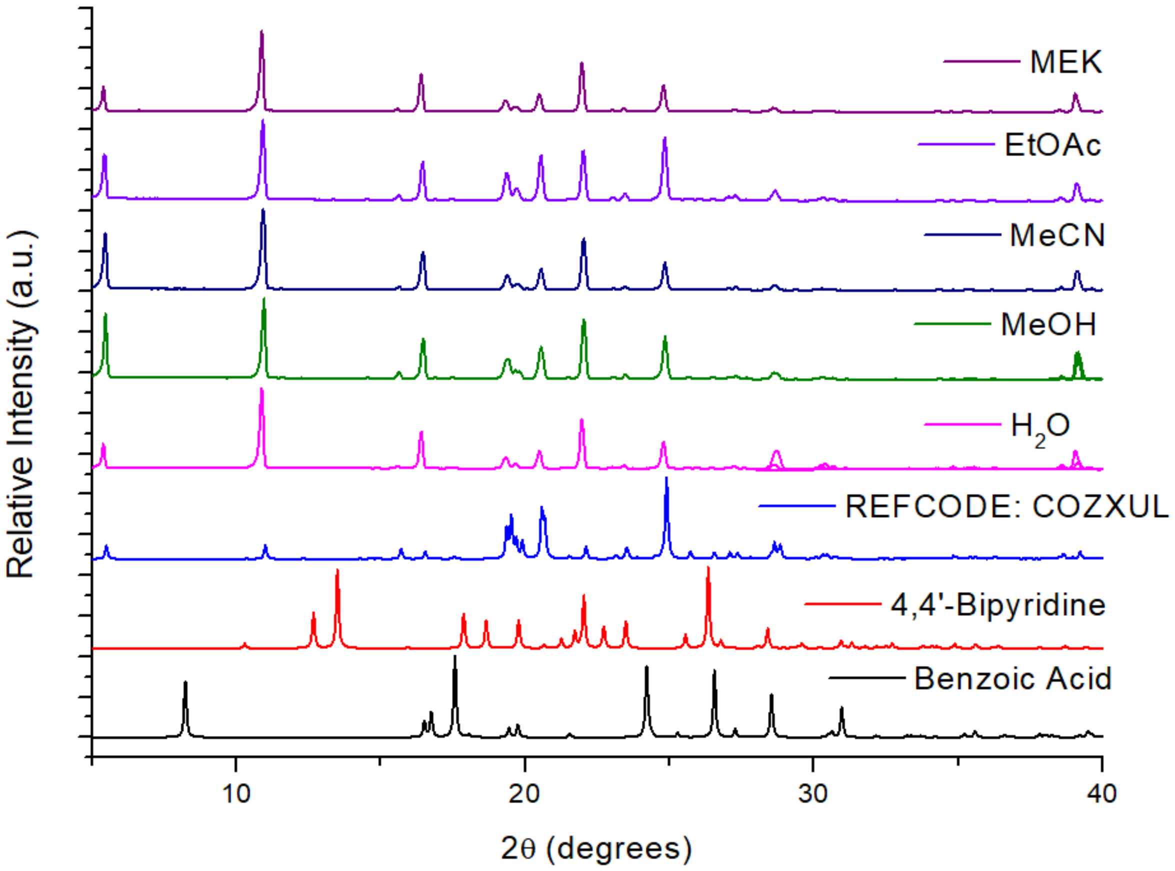

***Figure S3**. PXRD patterns from slurries in (top) MEK, EtOAc, MeCN, MeOH, water, cocrystal 3 calculated PXRD and the two starting materials.*

Slurry 4: Naproxen · Trans-1,2-bis(4-pyridyl)ethylene (2:1) [REFCODE: COZYAS]

Naproxen (76 mg, 0.33 mmol) and trans-1,2-bis(4-pyridyl)ethylene (30 mg, 0.17 mmol) were added to 0.5 mL deionized water and left to stir for 48 hours at 30°C. Naproxen (150 mg, 0.65 mmol) and trans-1,2-bis(4-pyridyl)ethylene (58 mg, 0.33 mmol) were added to 0.5 mL methanol and left to stir for 24 hours at room temperature. Naproxen (76 mg, 0.33 mmol) and trans-1,2-bis(4-pyridyl)ethylene (30 mg, 0.17 mmol) were added to 0.5 mL acetonitrile and left to stir for 24 hours at room temperature Naproxen (76 mg, 0.33 mmol) and trans-1,2-bis(4-pyridyl)ethylene (30 mg, 0.17 mmol) were added to 0.5 mL ethyl acetate and left to stir for 24 hours at room temperature. Naproxen (76 mg, 0.33 mmol) and trans-1,2-bis(4-pyridyl)ethylene (30 mg, 0.17 mmol) were added to 0.5 mL methyl ethyl ketone and left to stir for 24 hours at room temperature.

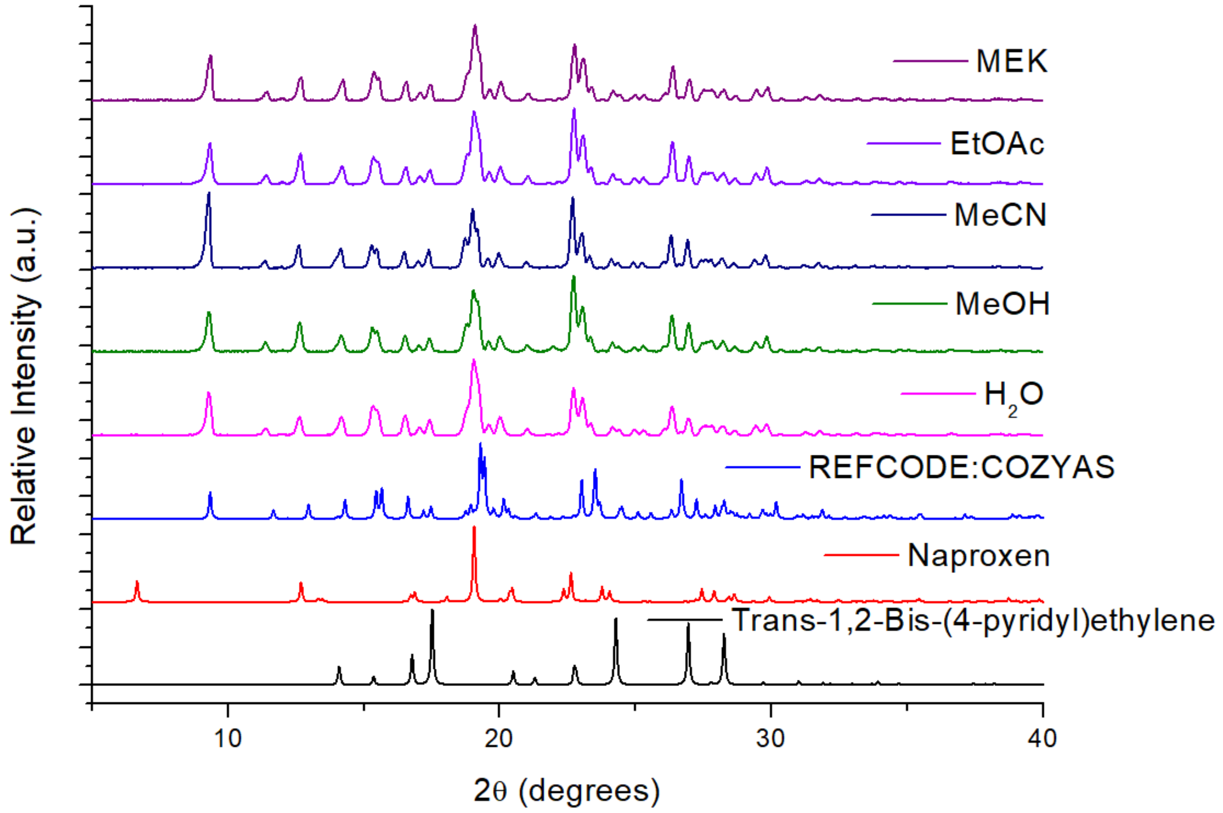

Figure S4. *PXRD patterns from slurries in (top) MEK, EtOAc, MeCN, MeOH, water, cocrystal 4 calculated PXRD and the two starting materials.*

Slurry 5: Glutaric acid · 1,2-bis(4-pyridyl)ethane (1:1) [REFCODE: COZYEW]

Glutaric acid (280 mg, 2.12 mmol) and 1,2-bis(4-pyridyl)ethane (390 mg, 2.12 mmol) were added to 1 mL deionized water and left to stir for 48 hours at 30°C. Glutaric acid (240 mg, 1.82 mmol) and 1,2-bis(4-pyridyl)ethane (344 mg, 1.82 mmol) were added to 0.75 mL methanol and left to stir for 24 hours at room temperature. Glutaric acid (100 mg, 0.76 mmol) and 1,2-bis(4-pyridyl)ethane (140 mg, 0.76 mmol) were added to 0.5 mL acetonitrile and left to stir for 24 hours at room temperature. Glutaric acid (350 mg, 2.65 mmol) and 1,2-bis(4-pyridyl)ethane (488 mg, 2.65mmol) were added to 1 mL ethyl acetate and left to stir for 24 hours at room temperature. Glutaric acid (75 mg, 0.57 mmol) and 1,2-bis(4-pyridyl)ethane (105 mg, 0.57 mmol) were added to 0.5 mL methyl ethyl ketone and left to stir for 24 hours at room temperature.

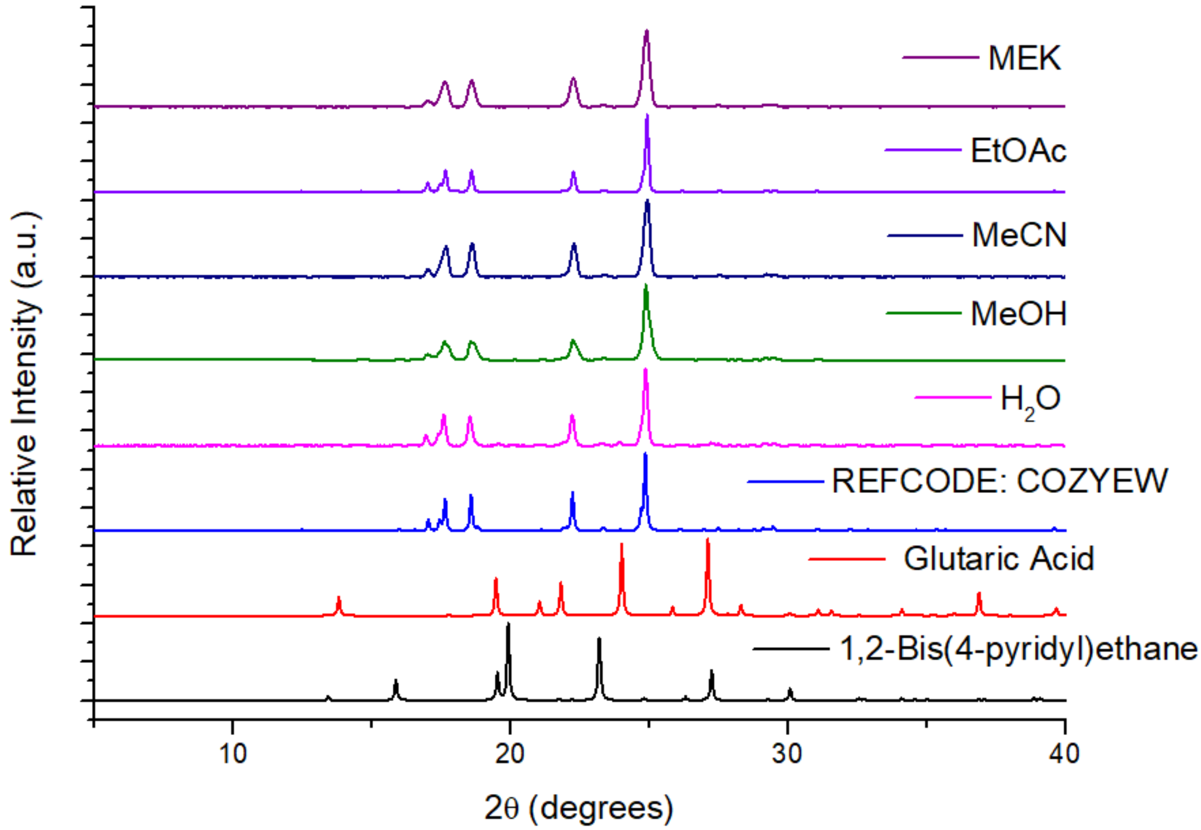

Figure S5. *PXRD patterns from slurries in (top) MEK, EtOAc, MeCN, MeOH, water, cocrystal 5 calculated PXRD and the two starting materials.*

Slurry 6: Glutaric acid · Trans-1,2-bis(4-pyridyl)ethylene (1:1) [REFCODE: COZYIA]

Glutaric acid (280 mg, 2.12 mmol) and trans-1,2-bis(4-pyridyl)ethylene (390 mg, 2.12 mmol) were added to 0.75 mL deionized water and left to stir for 48 hours at 30°C. Glutaric acid (240 mg, 1.82 mmol) and trans-1,2-bis(4-pyridyl)ethylene (340 mg, 1.82 mmol) were added to 0.75 mL methanol and left to stir for 24 hours at room temperature. Glutaric acid (95 mg, 0.72 mmol) and trans-1,2-bis(4-pyridyl)ethylene (60 mg, 0.72 mmol) were added to 0.5 mL acetonitrile and left to stir for 24 hours at room temperature. Glutaric acid (350 mg, 2.65 mmol) and trans-1,2-bis(4-pyridyl)ethylene (488mg, 2.65 mmol) were added to 1 mL ethyl acetate and left to stir for 24 hours at room temperature. Glutaric acid (140 mg, 1.05 mmol) and trans-1,2-bis(4-pyridyl)ethylene (188 mg, 1.05 mmol) were added to 0.5 mL methyl ethyl ketone and left to stir for 24 hours at room temperature.

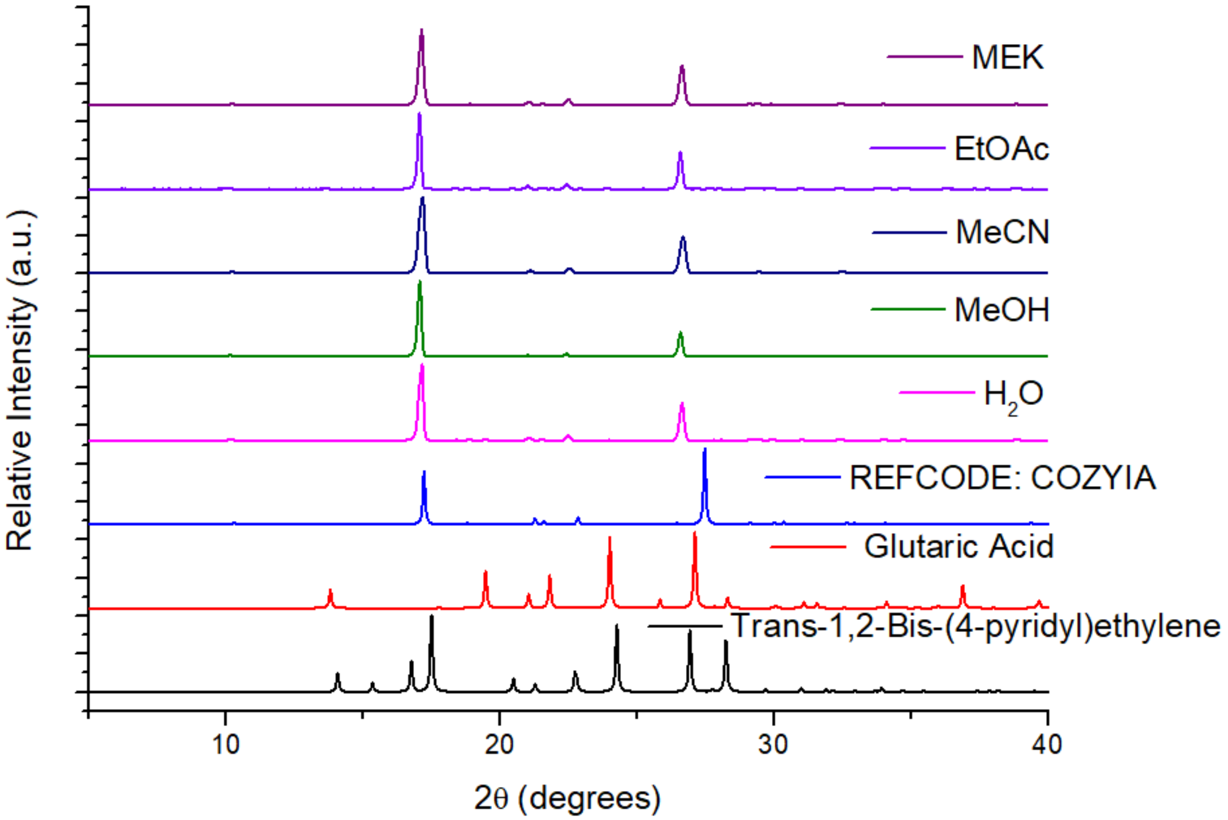

***Figure S6**. PXRD patterns from slurries in (top) MEK, EtOAc, MeCN, MeOH, water, cocrystal 6 calculated PXRD and the two starting materials.*

Slurry 7: Oxalic acid · Tetramethylpyrazine (1:1) [REFCODE: COZZOH]

Oxalic acid (40 mg, 0. 44 mmol) and tetramethylpyrazine (60 mg, 0.44 mmol) were added to 0.5 mL deionized water and left to stir for 48 hours at room temperature. Oxalic acid (100 mg, 1.11 mmol) and tetramethylpyrazine (152 mg, 1.11 mmol) were added to 0.5 mL methanol and left to stir for 24 hours at room temperature. Oxalic acid (100 mg, 1.11 mmol) and tetramethylpyrazine (152 mg, 1.11 mmol) were added to 0.5 mL acetonitrile and left to stir for 24 hours at room temperature. Oxalic acid (100 mg, 1.11 mmol) and tetramethylpyrazine (152 mg, 1.11 mmol) were added to 0.5 mL ethyl acetate and left to stir for 24 hours at room temperature. Oxalic acid (100 mg, 1.11 mmol) and tetramethylpyrazine (152 mg, 1.11 mmol) were added to 0.5 mL methyl ethyl ketone and left to stir for 24 hours at room temperature.

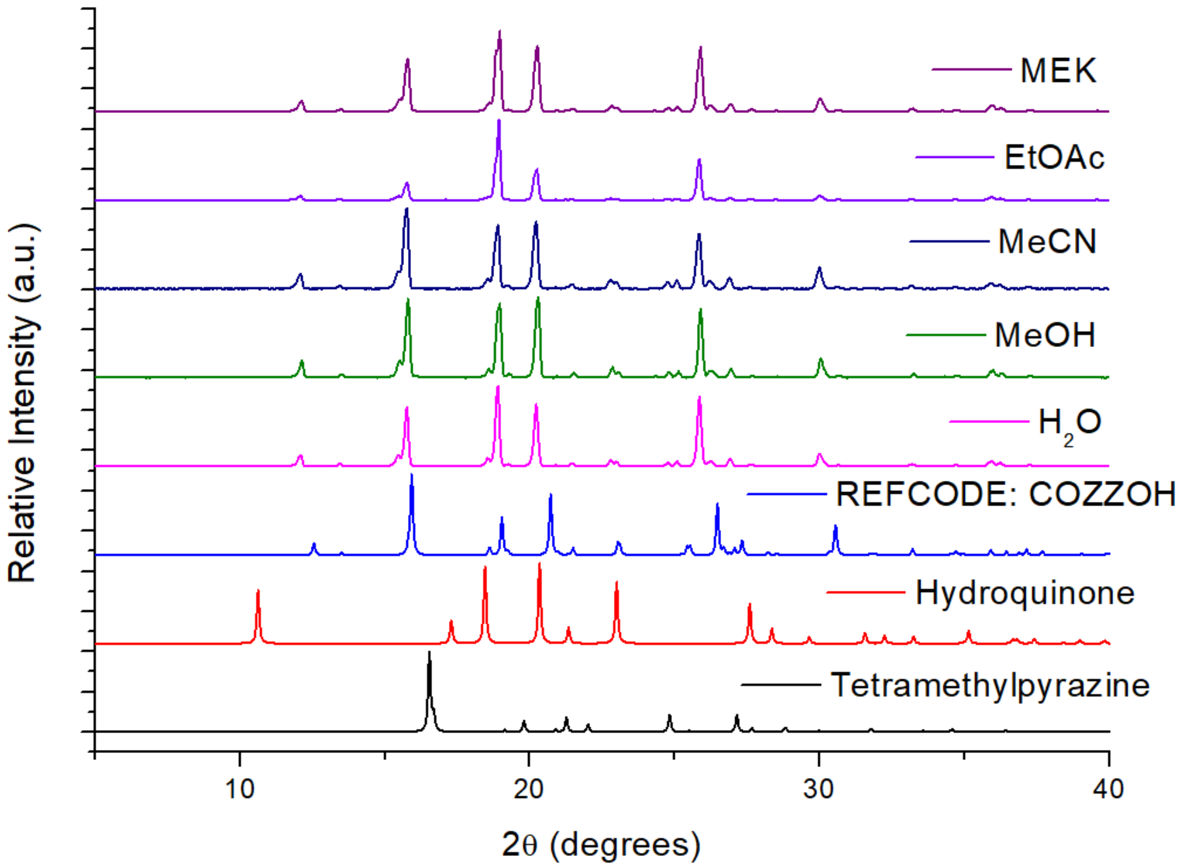

Figure S7. *PXRD patterns from slurries in (top) MEK, EtOAc, MeCN, MeOH, water, cocrystal 7 calculated PXRD and the two starting materials.*

Slurry 8: Isophthalic acid · 1,2-bis(4-pyridyl)ethane (1:1) [REFCODE: COZYUM]

Isophthalic acid (50 mg, 0.30 mmol) and 1,2-bis(4-pyridyl)ethane (55 mg, 0.30 mmol) were added to 0.5 mL deionized water and left to stir for 48 hours at room temperature. Isophthalic acid (226 mg, 1.36 mmol) and 1,2-bis(4-pyridyl)ethane (250 mg, 1.36 mmol) were added to 0.75 mL methanol and left to stir for 24 hours at room temperature. Isophthalic acid (100 mg, 0.60 mmol) and 1,2-bis(4-pyridyl)ethane (110 mg, 0.60 mmol) were added to 0.5 mL acetonitrile and left to stir for 24 hours at room temperature. Isophthalic acid (50 mg, 0.30 mmol) and 1,2-bis(4-pyridyl)ethane (55 mg, 0.30 mmol) were added to 0.5 mL ethyl acetate and left to stir for 24 hours at room temperature. Isophthalic acid (157 mg, 0.95 mmol) and 1,2-bis(4-pyridyl)ethane (175 mg, 0.95 mmol) were added to 0.5 mL methyl ethyl ketone and left to stir for 24 hours at room temperature.

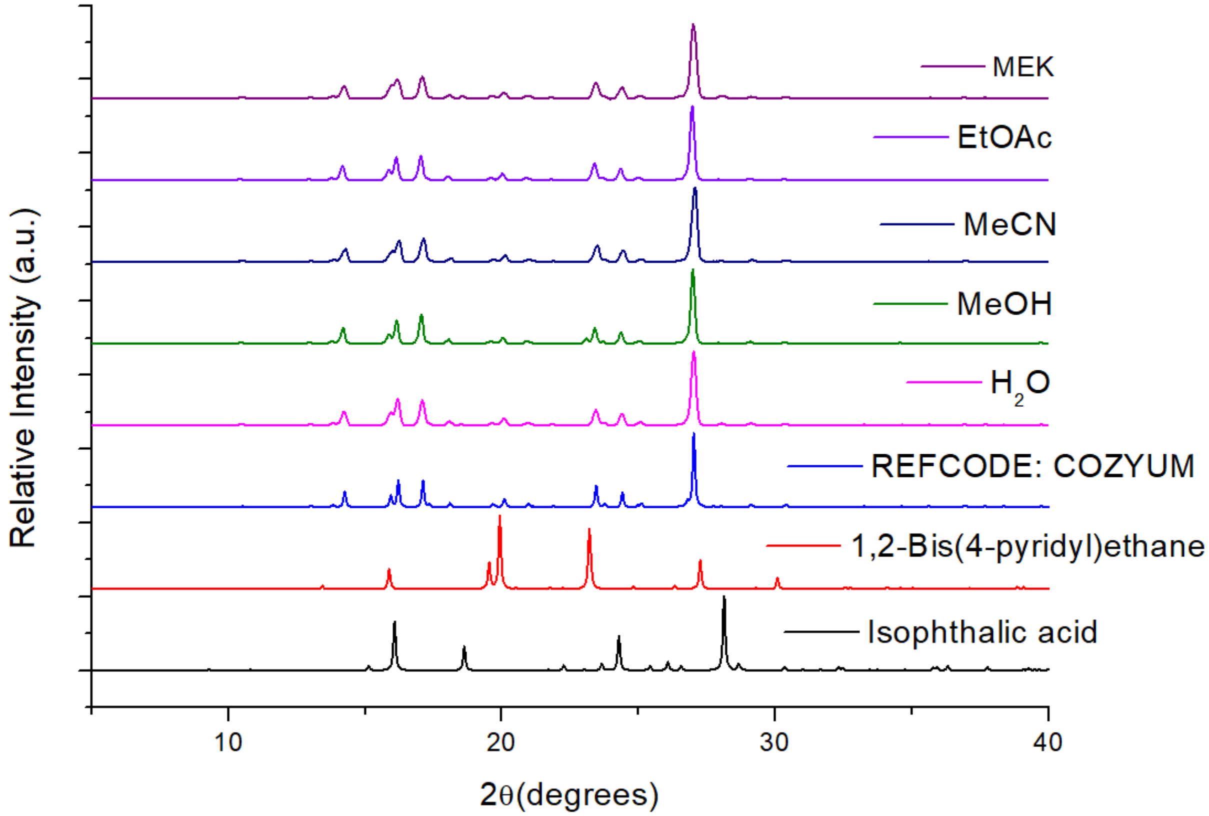

Figure S8. *PXRD patterns from slurries in (top) MEK, EtOAc, MeCN, MeOH, water, cocrystal 8 calculated PXRD and the two starting materials.*

Slurry 9: Trimesic acid · 1,2-Bis(4-pyridyl)ethane (1:2) [REFCODE: COZXEV]

Trimesic acid (35 mg, 0.30 mmol) and 1,2-bis(4-pyridyl)ethane (60 mg, 0.60 mmol) were added to 0.5 mL deionized water and left to stir for 48 hours at 30°C. Trimesic acid (125 mg, 0.60 mmol) and 1,2-bis(4-pyridyl)ethane (220 mg, 1.20 mmol) were added to 0.5 mL methanol and left to stir for 24 hours at room temperature. Trimesic acid (46 mg, 0.22 mmol) and 1,2-bis(4-pyridyl)ethane (80 mg, 0.43 mmol) were added to 0.5 mL acetonitrile and left to stir for 24 hours at room temperature. Trimesic acid (46 mg, 0.22 mmol) and 1,2-bis(4-pyridyl)ethane (80 mg, 0.43 mmol) were added to 0.5 mL ethyl acetate and left to stir for 24 hours at room temperature. Trimesic acid (46 mg, 0.22 mmol) and 1,2-bis(4-pyridyl)ethane (80 mg, 0.43 mmol) were added to 0.5 mL methyl ethyl ketone and left to stir for 24 hours at room temperature.

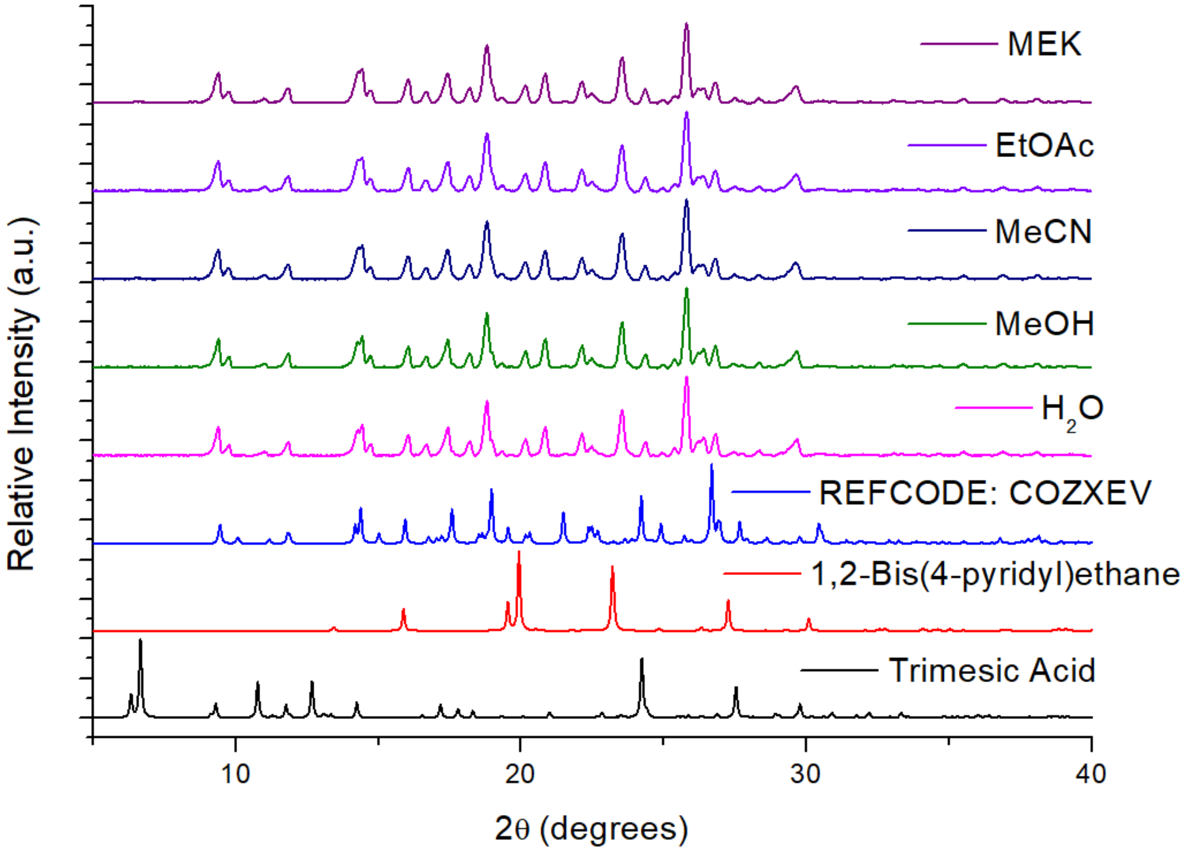

Figure S9. *PXRD patterns from slurries in (top) MEK, EtOAc, MeCN, MeOH, water, cocrystal 9 calculated PXRD and the two starting materials.*

Slurry 10: 1-Naphthol · 1,2-bis(4-pyridyl)ethane (2:1) [REFCODE: COZZAT]

1-Naphthol (50 mg, 0.35 mmol) and 1,2-bis(4-pyridyl)ethane (32 mg, 0.18 mmol) were added to 0.5 mL deionized water and left to stir for 48 hours at room temperature. 1-Naphthol (344 mg, 2.39 mmol) and 1, 2-bis(4-pyridyl)ethane (220 mg, 1.20 mmol) were added to 0.75 mL methanol and left to stir for 24 hours at room temperature. 1-Naphthol (150 mg, 1.04 mmol) and 1, 2-bis(4-pyridyl)ethane (96 mg, 0.52 mmol) were added to 0.5 mL acetonitrile and left to stir for 24 hours at room temperature. 1-Naphthol (100 mg, 0.69 mmol) and 1, 2-bis(4-pyridyl)ethane (64 mg, 0.35 mmol) were added to 0.5 mL ethyl acetate and left to stir for 24 hours at room temperature. 1-Naphthol (200 mg, 1.38 mmol) and 1, 2-bis(4-pyridyl)ethane (128 mg, 0.69 mmol) were added to 0.5 mL methyl ethyl ketone and left to stir for 24 hours at room temperature.

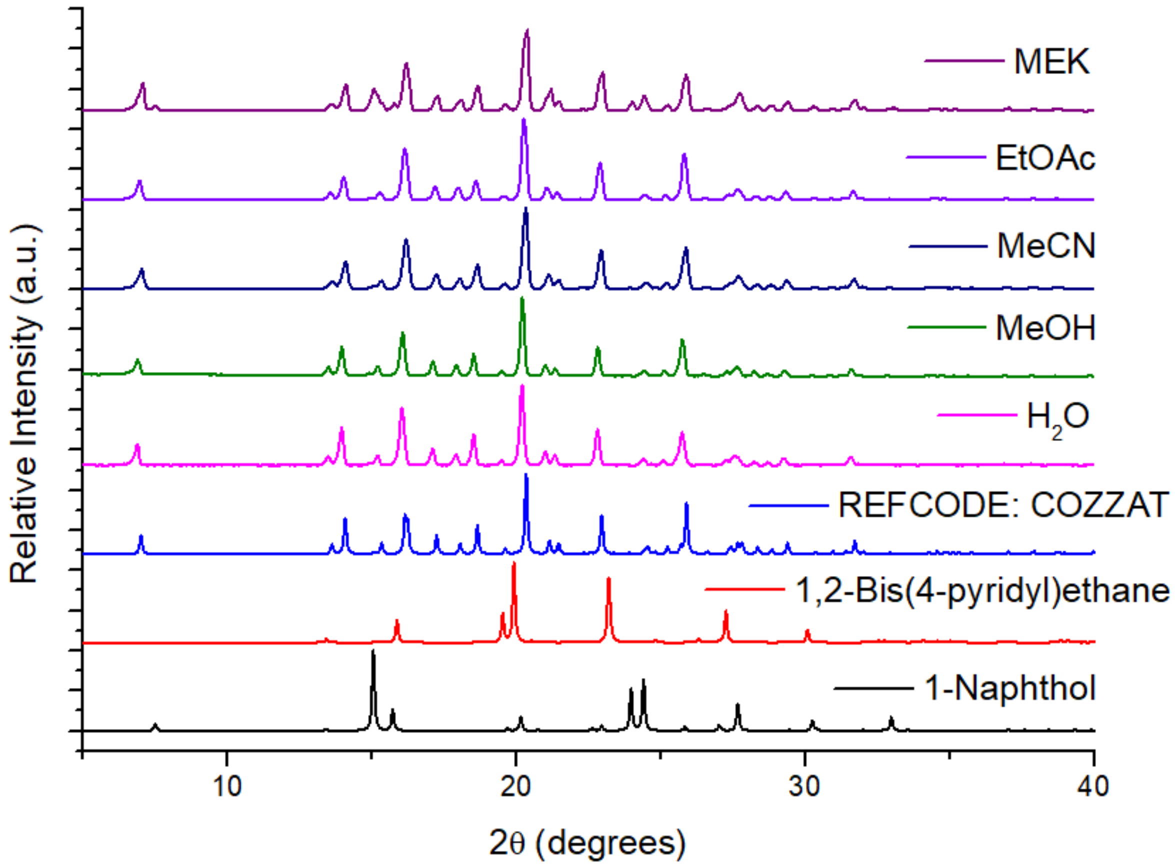

Figure S10. *PXRD patterns from slurries in (top) MEK, EtOAc, MeCN, MeOH, water, cocrystal 10 calculated PXRD and the two starting materials.*

Slurry 11: 1-Naphthol · trans-1,2-bis(4-pyridyl)ethylene (2:1) [REFCODE: QOVHOV]

1-Naphthol (50 mg, 0.35 mmol) and trans-1,2-bis(4-pyridyl)ethylene (32 mg, 0.18 mmol) were added to 0.5 mL deionized water and left to stir for 48 hours at room temperature. 1-Naphthol (250 mg, 1.74 mmol) and trans-1,2-bis(4-pyridyl)ethylene (158 mg, 0.87 mmol) were added to 0.5 mL methanol and left to stir for 24 hours at room temperature. 1-Naphthol (100 mg, 1.69 mmol) and trans-1,2-bis(4-pyridyl)ethylene (65 mg, 0.85 mmol) were added to 0.5 mL acetonitrile and left to stir for 24 hours at room temperature. 1-Naphthol (150 mg, 1.04 mmol) and trans-1,2-bis(4-pyridyl)ethylene (96 mg, 0.52 mmol) were added to 0.5 mL ethyl acetate and left to stir for 24 hours at room temperature. 1-Naphthol (200 mg, 1.38 mmol) and trans-1,2-bis(4-pyridyl)ethylene (128 mg, 0.69 mmol) were added to 0.5 mL methyl ethyl ketone and left to stir for 24 hours at room temperature.

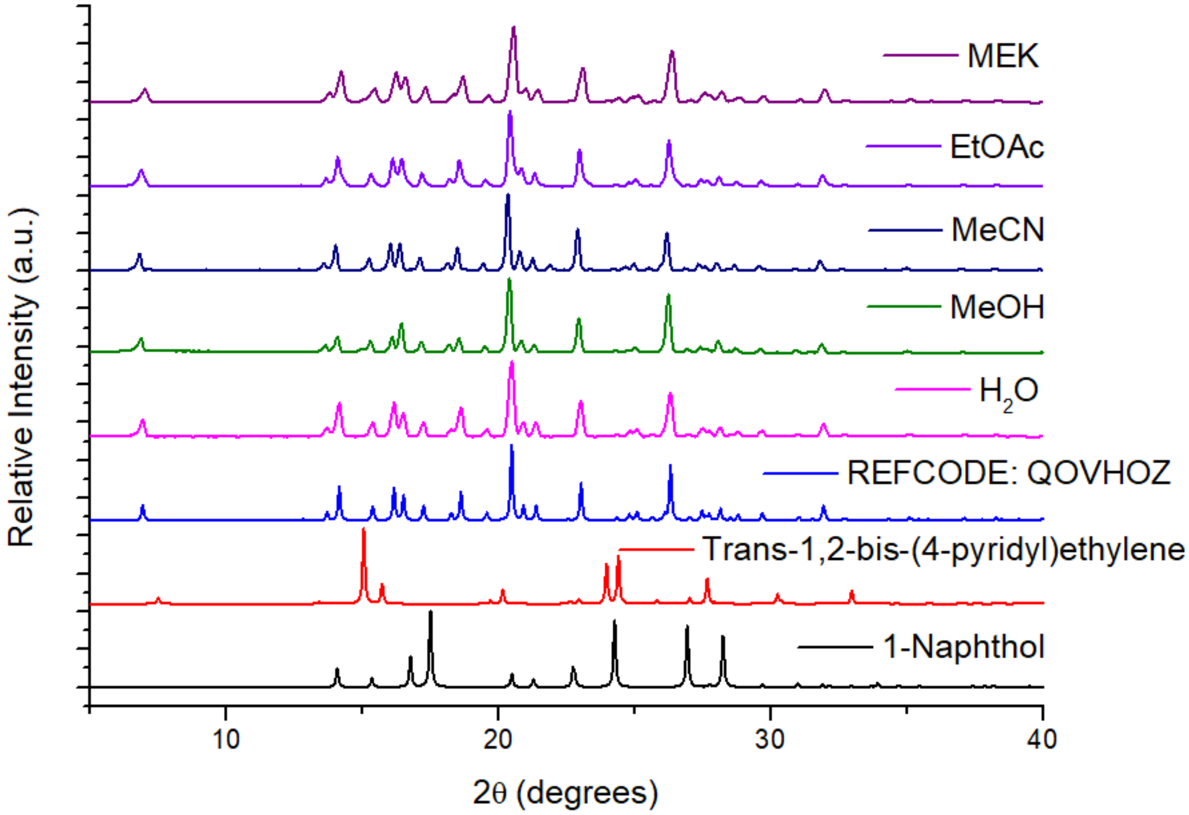

Figure S11. *PXRD patterns from slurries in (top) MEK, EtOAc, MeCN, MeOH, water, cocrystal 11 calculated PXRD and the two starting materials.*

Slurry 12: 4,4′-Biphenol · 1,2-bis(4-pyridyl)ethane (1:1) [REFCODE: COZZEX]

4,4′-Biphenol (50 mg, 0.27 mmol) and 1,2-bis(4-pyridyl)ethane (50 mg, 0.27 mmol) were added to 0.5 mL deionized water and left to stir for 48 hours at room temperature. 4,4′-Biphenol (220 mg, 1.20 mmol) and 1, 2-bis(4-pyridyl)ethane (220 mg, 1.20 mmol) were added to 0.75 mL methanol and left to stir for 24 hours at room temperature. 4,4′-Biphenol (100 mg, 0.54 mmol) and 1, 2-bis(4-pyridyl)ethane (100 mg, 0.54 mmol) were added to 0.5 mL acetonitrile and left to stir for 24 hours at room temperature. 4,4′-Biphenol (100 mg, 0.54 mmol) and 1, 2-bis(4-pyridyl)ethane (100 mg, 0.54 mmol) were added to 0.5 mL ethyl acetate and left to stir for 24 hours at room temperature. 4,4′-Biphenol (100 mg, 0.54 mmol) and 1, 2-bis(4-pyridyl)ethane (100 mg, 0.54 mmol) were added to 0.5 mL methyl ethyl ketone and left to stir for 24 hours at room temperature.

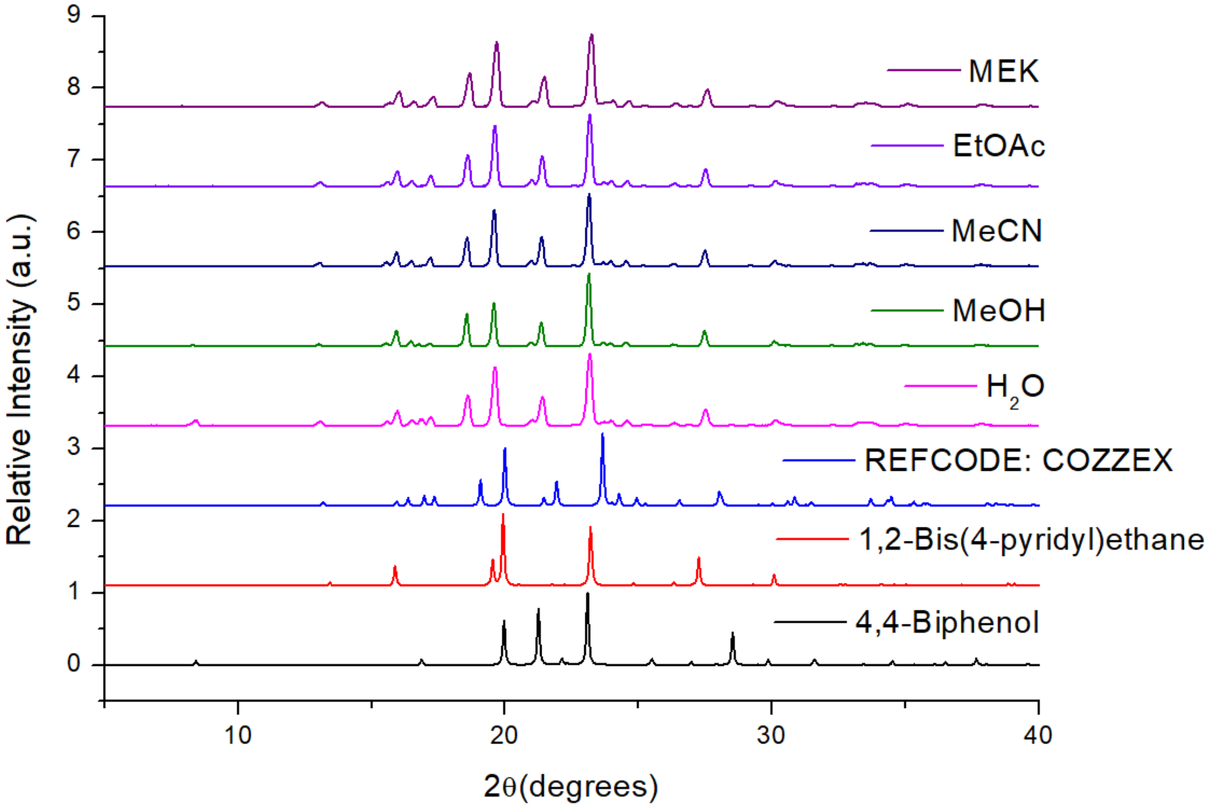

Figure S12. *PXRD patterns from slurries in (top) MEK, EtOAc, MeCN, MeOH, water, cocrystal 12 calculated PXRD and the two starting materials.*

Slurry 13: 4,4′-Biphenol · Trans-1,2-bis(4-pyridyl)ethylene (1:1) [REFCODE: QOVHUF]

4,4′-Biphenol (50 mg, 0.27 mmol) and trans-1,2-bis(4-pyridyl)ethylene (50 mg, 0.27 mmol) were added to 0.5 mL deionized water and left to stir for 48 hours at 30°C. 4,4′-Biphenol (220 mg, 1.20 mmol) and trans-1,2-bis(4-pyridyl)ethylene (220 mg, 1.20 mmol) were added to 0.5 mL methanol and left to stir for 24 hours at room temperature. 4,4′-Biphenol (50 mg, 0.27 mmol) and trans-1,2-bis(4-pyridyl)ethylene (50 mg, 0.27 mmol) were added to 0.5 mL acetonitrile and left to stir for 24 hours at room temperature. 4,4′-Biphenol (50 mg, 0.27 mmol) and trans-1,2-bis(4-pyridyl)ethylene (50 mg, 0.27 mmol) were added to 0.5 mL ethyl acetate and left to stir for 24 hours at room temperature. 4,4′-Biphenol (50 mg, 0.27 mmol) and trans-1,2-bis(4-pyridyl)ethylene (50 mg, 0.27 mmol) were added to 0.5 mL methyl ethyl ketone and left to stir for 24 hours at room temperature.

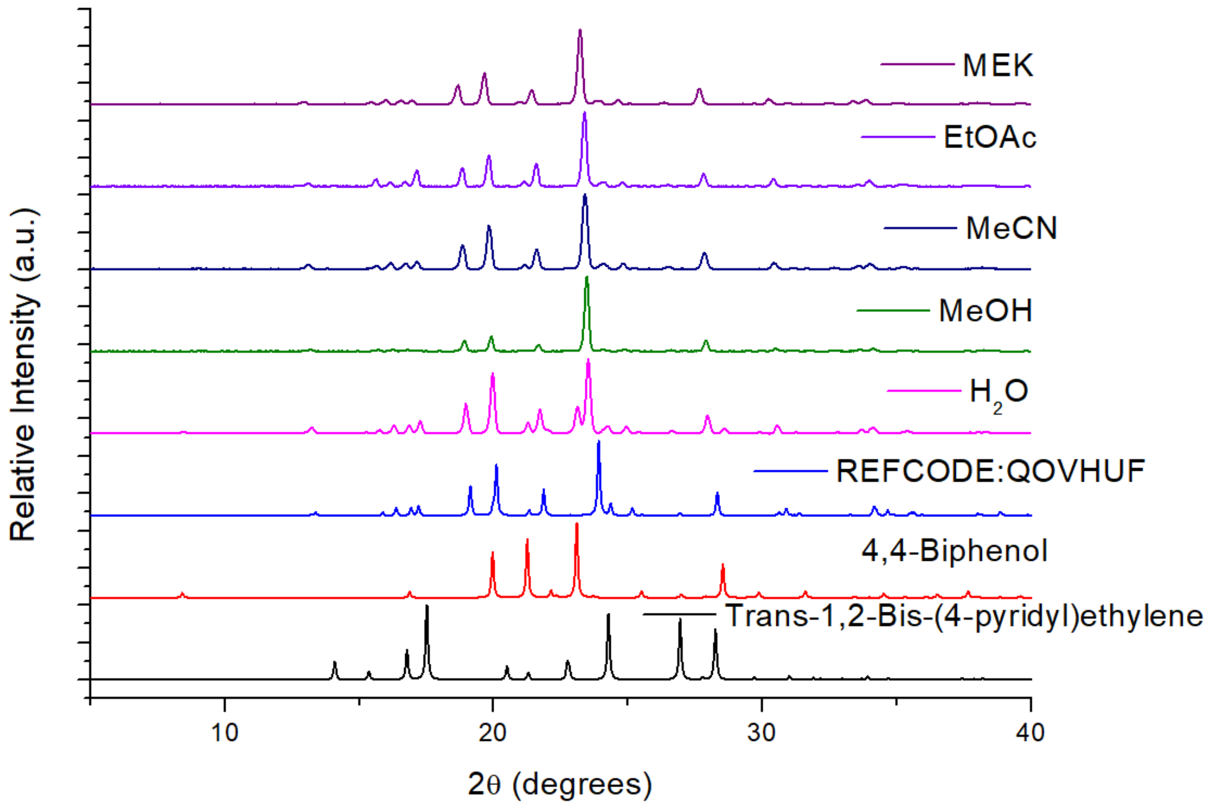

Figure S13. *PXRD patterns from slurries in (top) MEK, EtOAc, MeCN, MeOH, water, cocrystal 13 calculated PXRD and the two starting materials.*

Slurry 14: Hydroquinone · Trans-1,2-bis(4-pyridyl)ethylene (1:1) [REFCODE: COZZIB]

Hydroquinone (70 mg, 0.64 mmol) and trans-1,2-bis(4-pyridyl)ethylene (118 mg, 0.64 mmol) were added to 0.5 mL deionized water and left to stir for 48 hours at 30°C. Hydroquinone (91 mg, 0.83 mmol) and trans-1,2-bis(4-pyridyl)ethylene (150 mg, 0.82 mmol) were added to 0.5 mL methanol and left to stir for 24 hours at room temperature. Hydroquinone (85 mg, 0.77 mmol) and trans-1,2-bis(4-pyridyl)ethylene (142 mg, 0.77 mmol) were added to 0.5 mL acetonitrile and left to stir for 24 hours at room temperature. Hydroquinone (100 mg, 0.91 mmol) and trans-1,2-bis(4-pyridyl)ethylene (167 mg, 0.91 mmol) were added to 0.5 mL ethyl acetate and left to stir for 24 hours at room temperature. Hydroquinone (150 mg, 1.36 mmol) and trans-1,2-bis(4-pyridyl)ethylene (250 mg, 1.36 mmol) were added to 0.8 mL methyl ethyl ketone and left to stir for 24 hours at room temperature.

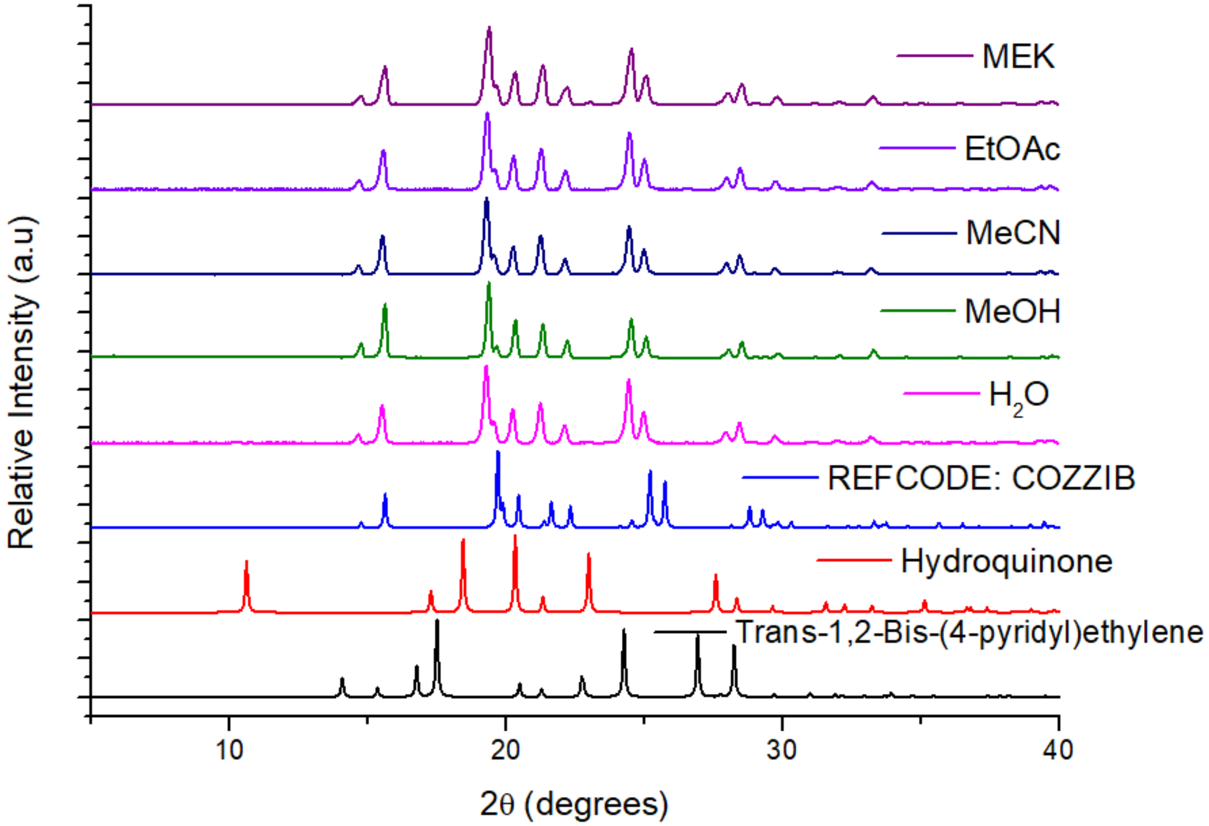

Figure S14. *PXRD patterns from slurries in (top) MEK, EtOAc, MeCN, MeOH, water, cocrystal 14 calculated PXRD and the two starting materials.*

Slurry 15: Hydroquinone · Tetramethylpyrazine (1:1) [REFCODE: COZZOH]

Hydroquinone (70 mg, 0.63 mmol) and tetramethylpyrazine (86 mg, 0.63 mmol) were added to 0.5 mL deionized water and left to stir for 48 hours at 30°C. Hydroquinone (150 mg, 1.36 mmol) and tetramethylpyrazine (185 mg, 1.36 mmol) were added to 0.5 mL methanol and left to stir for 24 hours at room temperature. Hydroquinone (100 mg, 0.91 mmol) and tetramethylpyrazine (123 mg, 0.91 mmol) were added to 0.5 mL acetonitrile and left to stir for 24 hours at room temperature. Hydroquinone (100 mg, 0.91 mmol) and tetramethylpyrazine (123 mg, 0.91 mmol) were added to 0.5 mL ethyl acetate and left to stir for 24 hours at room temperature. Hydroquinone (150 mg, 1.36 mmol) and tetramethylpyrazine (185 mg, 1.36 mmol) were added to 0.5 mL methyl ethyl ketone and left to stir for 24 hours at room temperature.

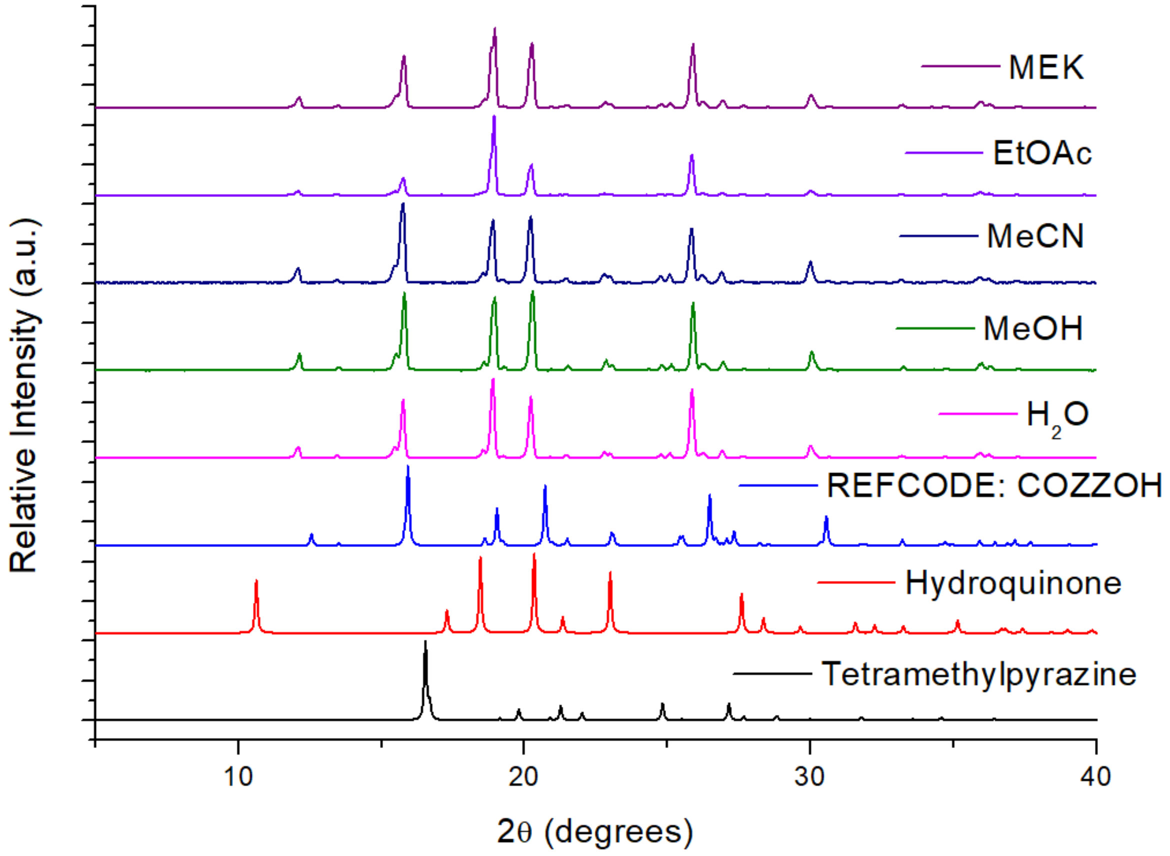

Figure S15. *PXRD patterns from slurries in (top) MEK, EtOAc, MeCN, MeOH, water, cocrystal 15 calculated PXRD and the two starting materials.*

Slurry 16: Resorcinol · Tetramethylpyrazine (2:3) [REFCODE: COZZUN]

Resorcinol (300 mg, 2.72 mmol) and tetramethylpyrazine (556 mg, 4.09 mmol) were added to 1.5 mL deionized water and left to stir for 48 hours at room temperature. Resorcinol (300 mg, 2.72 mmol) and tetramethylpyrazine (556 mg, 4.09 mmol) were added to 1.5 mL methanol and left to stir for 24 hours at room temperature. Resorcinol (300 mg, 2.72 mmol) and tetramethylpyrazine (556 mg, 4.09 mmol) were added to 1.5 mL acetonitrile and left to stir for 24 hours at room temperature. Resorcinol (300 mg, 2.72 mmol) and tetramethylpyrazine (556 mg, 4.09 mmol) were added to 1.5 mL ethyl acetate and left to stir for 24 hours at room temperature. Resorcinol (300 mg, 2.72 mmol) and tetramethylpyrazine (556 mg, 4.09 mmol) were added to 1.5 mL methyl ethyl ketone and left to stir for 24 hours at room temperature.

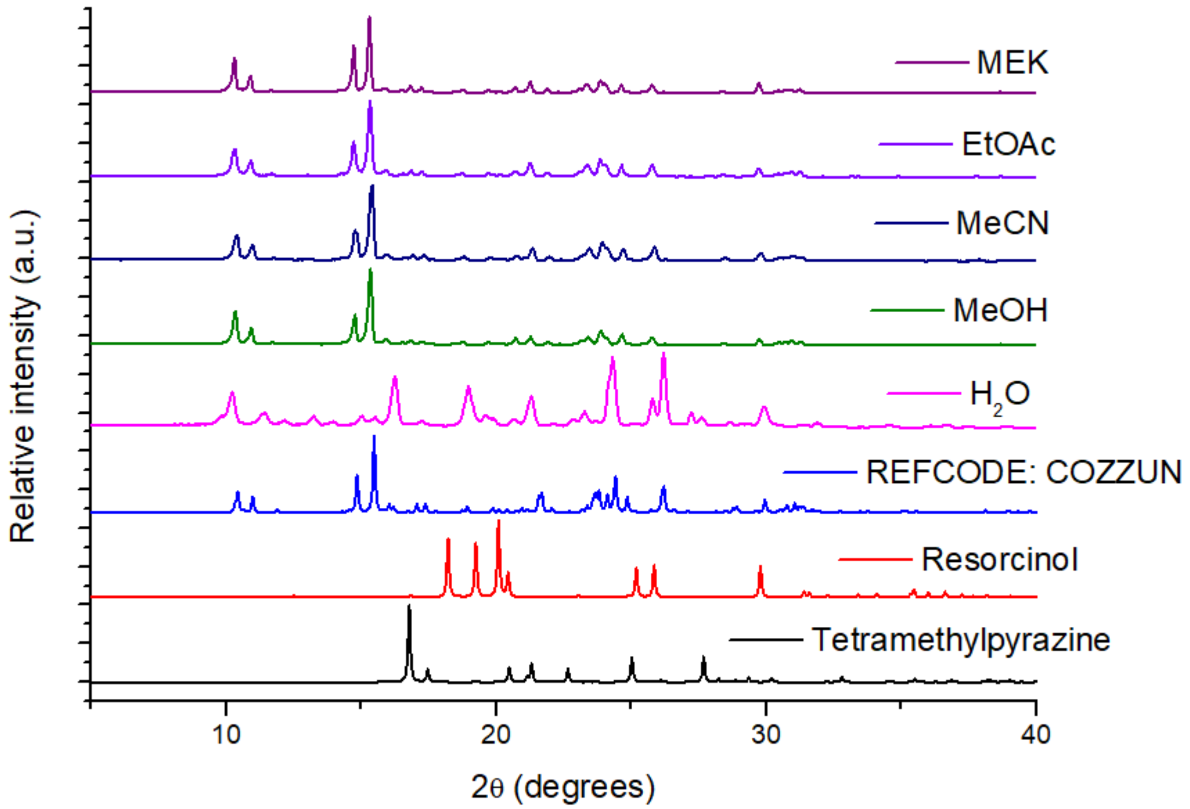

Figure S16. *PXRD patterns from slurries in (top) MEK, EtOAc, MeCN, MeOH, water, cocrystal 16 calculated PXRD and the two starting materials.*

Slurry 17: 2,7-Dihydroxynaphthalene · Tetramethylpyrazine (1:2) [REFCODE: CUBBAD]

2,7-Dihydroxynaphthalene (30 mg, 0.19 mmol) and tetramethylpyrazine (50 mg, 0.37 mmol) were added to 0.5 mL deionized water and left to stir for 48 hours at room temperature. 2,7-Dihydroxynaphthalene (240 mg, 1.50 mmol) and tetramethylpyrazine (204 mg, 1.50 mmol) were added to 0.8 mL methanol and left to stir for 24 hours at room temperature. 2,7-Dihydroxynaphthalene (88 mg, 0.65 mmol) and tetramethylpyrazine (150 mg, 1.10 mmol) were added to 0.5 mL acetonitrile and left to stir for 24 hours at room temperature. 2,7-Dihydroxynaphthalene (88 mg, 0.65 mmol) and tetramethylpyrazine (150 mg, 1.10 mmol) were added to 0.5 mL ethyl acetate and left to stir for 24 hours at room temperature. 2,7-Dihydroxynaphthalene (88 mg, 0.65 mmol) and tetramethylpyrazine (150 mg, 1.10 mmol) were added to 0.5 mL methyl ethyl ketone and left to stir for 24 hours at room temperature.

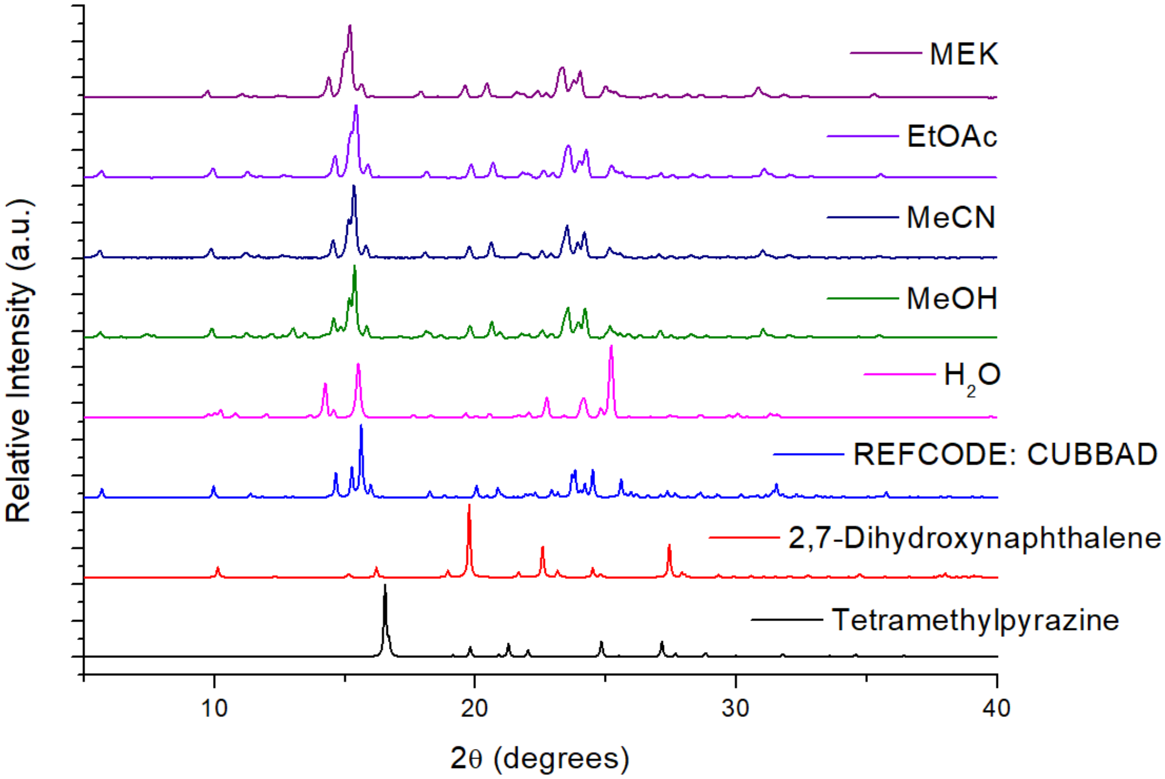

Figure S17. *PXRD patterns from slurries in (top) MEK, EtOAc, MeCN, MeOH, water, cocrystal 17 calculated PXRD and the two starting materials.*

Slurry 18: 4, 4'-Bipyridine · Carbamazepine (1:2) [REFCODE: XAQQUC]

4, 4'-Bipyridine (33 mg, 0.21 mmol) and carbamazepine (100 mg, 0.42 mmol) were added to 0.5 mL deionized water and left to stir for 48 hours at room temperature. 4, 4'-Bipyridine (120 mg, 0.77 mmol) and carbamazepine (363 mg, 1.53 mmol) were added to 0.75 mL methanol and left to stir for 24 hours at room temperature. 4, 4'-Bipyridine (66 mg, 0.42 mmol) and carbamazepine (100 mg, 0.42 mmol) were added to 0.5 mL acetonitrile and left to stir for 24 hours at room temperature.4,4'-Bipyridine (79 mg, 0.51 mmol) and carbamazepine (120 mg, 0.51 mmol) were added to 0.5 mL ethyl acetate and left to stir for 24 hours at room temperature. 4, 4'-Bipyridine (125 mg, 0.8 mmol) and carbamazepine (200 mg, 0.84 mmol) were added to 0.6 mL methyl ethyl ketone and left to stir for 24 hours at room temperature.

1:2 ratios were unsuccessful, the carbamazepine hydrate was being produced hence the increase in 4,4-bipyridine concentration.

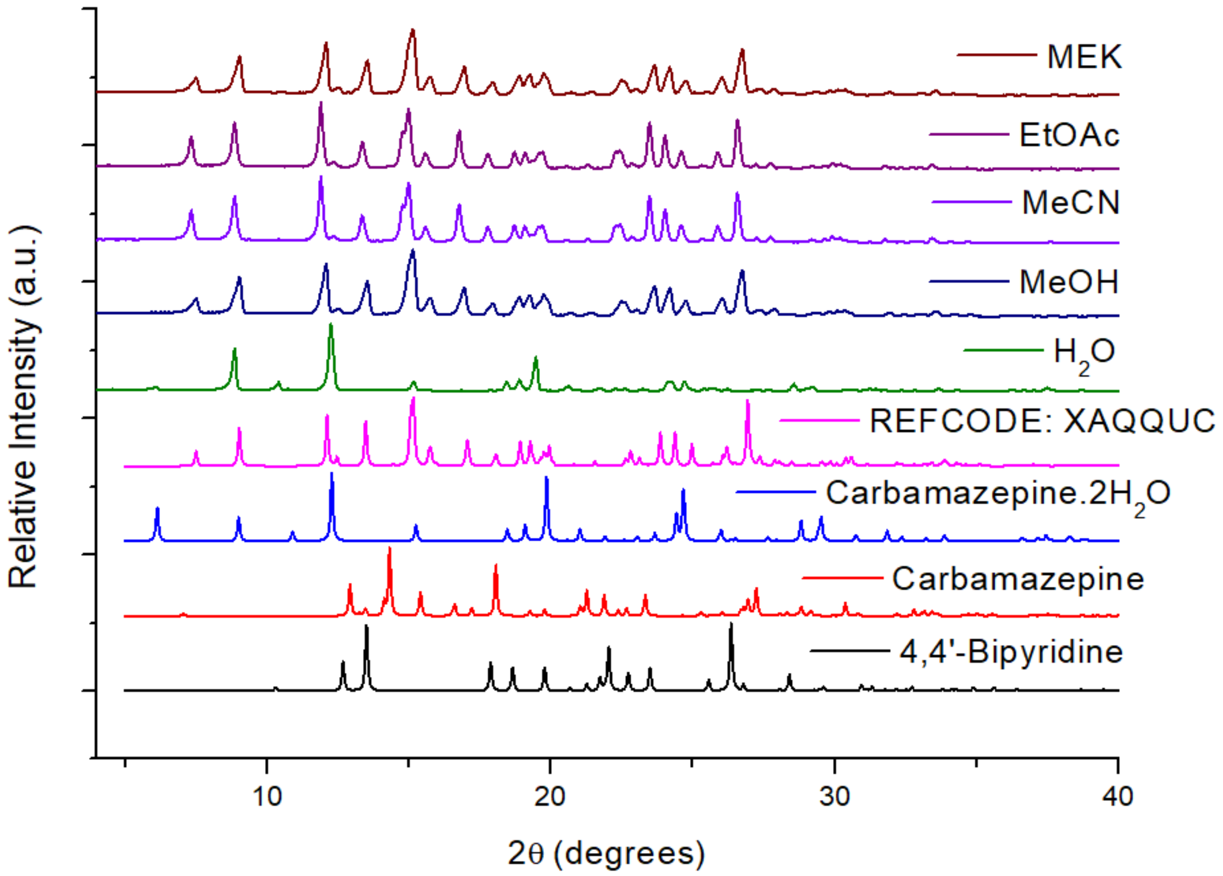

Figure S18. *PXRD patterns from slurries in (top) MEK, EtOAc, MeCN, MeOH, water, cocrystal 18 calculated PXRD and the two starting materials.*

Slurry 19: 4-Aminobenzoic acid · Carbamazepine (1:1) [REFCODE: XOXHEY]

4-Aminobenzoic acid (60 mg, 0.59 mmol) and carbamazepine (139 mg, 0.59 mmol) were added to 0.5 mL deionized water and left to stir for 48 hours at 30°C. 4-Aminobenzoic acid (100 mg, 0.73 mmol) and carbamazepine (172 mg, 0.73 mmol) were added to 1 mL methanol and left to stir for 24 hours at room temperature. 4-Aminobenzoic acid (60 mg, 0.59 mmol) and carbamazepine (139 mg, 0.59 mmol) were added to 0.5 mL acetonitrile and left to stir for 24 hours at room temperature. 4-Aminobenzoic acid (100 mg, 0.73 mmol) and carbamazepine (172 mg, 0.73 mmol) were added to 0.75 mL ethyl acetate and left to stir for 24 hours at room temperature. 4-Aminobenzoic acid (100 mg, 0.73 mmol) and carbamazepine (172 mg, 0.73 mmol) were added to 0.75 mL methyl ethyl ketone and left to stir for 24 hours at room temperature.

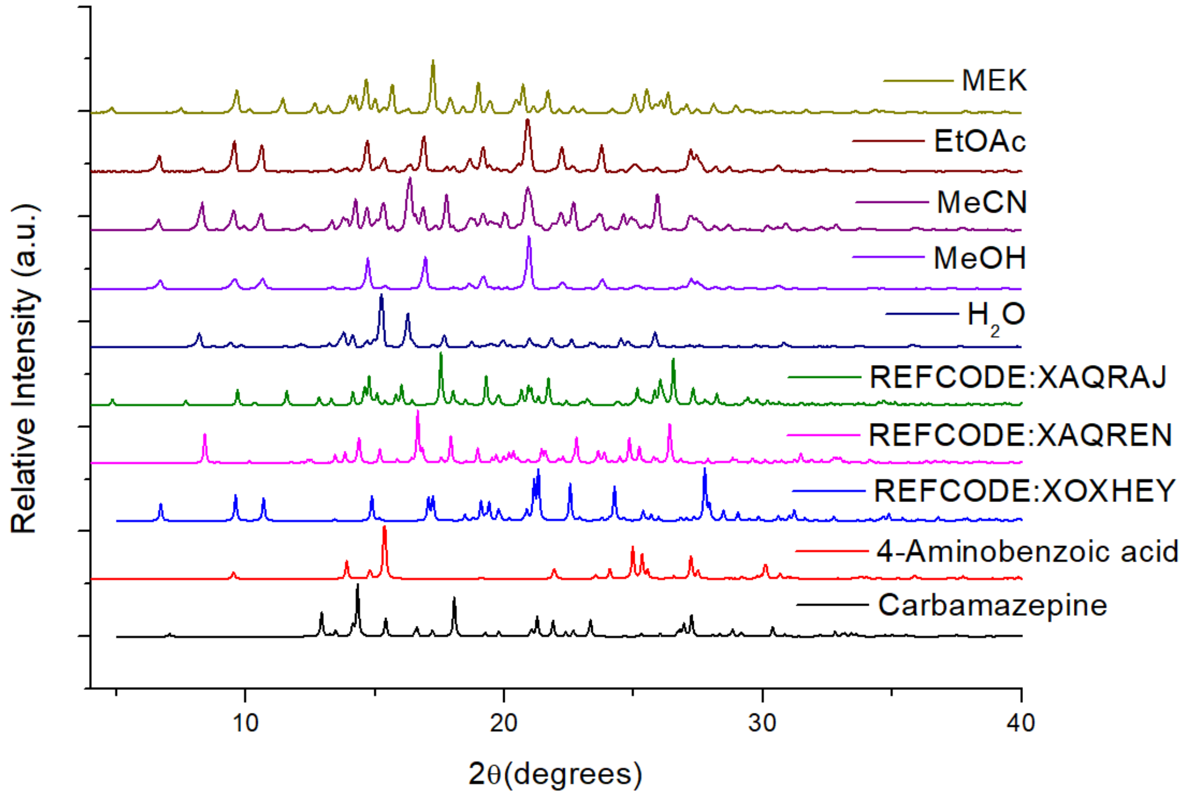

Figure S19. *PXRD patterns from slurries in (top) MEK, EtOAc, MeCN, MeOH, water, cocrystal 19 calculated PXRD 2:1, 2:1 hydrate, 1:1 stoichiometric cocrystals and the two starting materials.*

Slurry 20: 2, 6-Pyridinecarboxylic acid · Carbamazepine (1:1) [REFCODE: XAQRIR]

2,6-Pyridinedicarboxylic acid (40 mg, 0.24 mmol) and carbamazepine (56.4 mg, 0.24 mmol) were added to 0.5 mL deionized water and left to stir for 48 hours at 30°C. 2, 6-Pyridinedicarboxylic acid (40 mg, 0.24 mmol) and carbamazepine (56.4 mg, 0.24 mmol) were added to 0.5 mL methanol and left to stir for 24 hours at room temperature. 2, 6-Pyridinedicarboxylic acid (40 mg, 0.24 mmol) and carbamazepine (56.4 mg, 0.24 mmol) were added to 0.5 mL acetonitrile and left to stir for 24 hours at room temperature. 2, 6-Pyridinedicarboxylic acid (40 mg, 0.24 mmol) and carbamazepine (56.4 mg, 0.24 mmol) were added to 0.5 mL ethyl acetate and left to stir for 24 hours at room temperature. 2, 6-Pyridinedicarboxylic acid (40 mg, 0.24 mmol) and carbamazepine (56.4 mg, 0.24 mmol) were added to 0.5 mL methyl ethyl ketone and left to stir for 24 hours at room temperature.

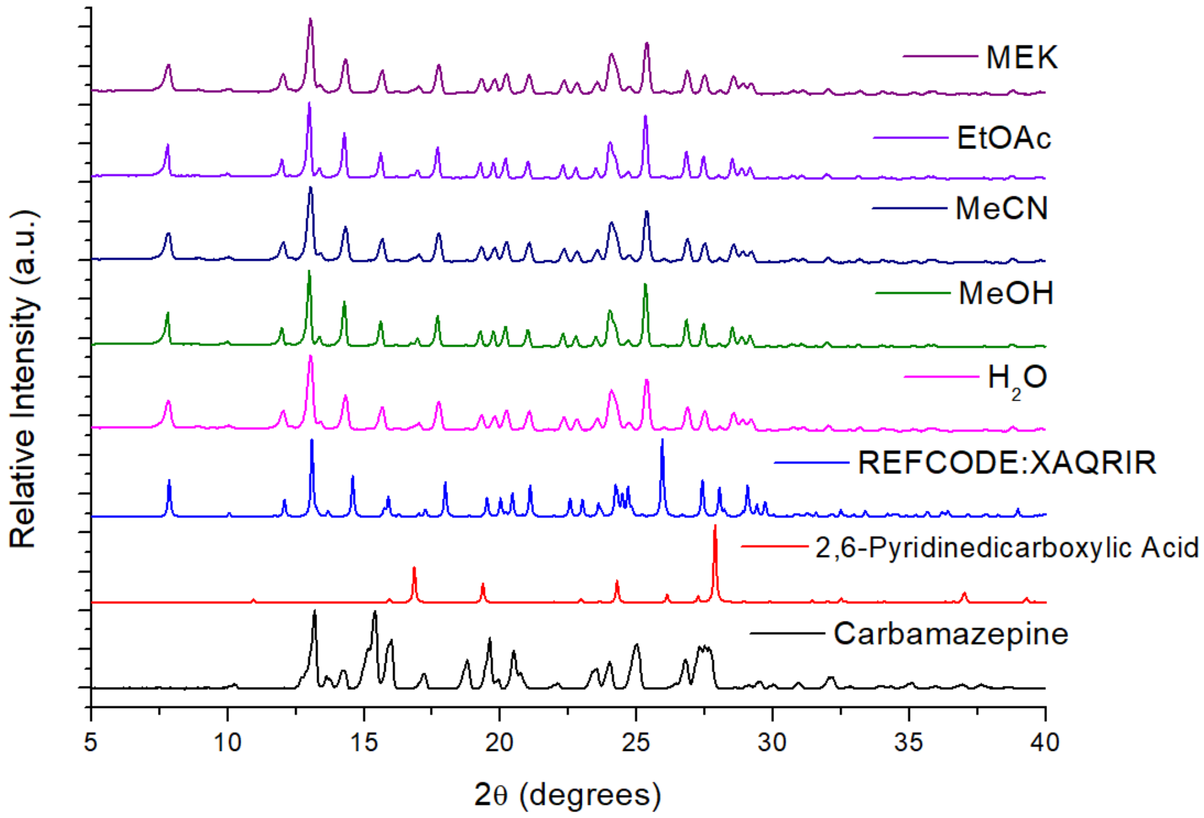

Figure S20. *PXRD patterns from slurries in (top) MEK, EtOAc, MeCN, MeOH, water, cocrystal 20 calculated PXRD and the two starting materials.*

Slurry 21: Benzoquinone · Carbamazepine (1:2) [REFCODE: UNEYOB]

Benzoquinone (20 mg, 0.19 mmol) and carbamazepine (88 mg, 0.37 mmol) were added to 0.5 mL deionized water and left to stir for 48 hours at 30°C. Benzoquinone (20 mg, 0.19 mmol) and carbamazepine (88 mg, 0.37 mmol) were added to methanol and left to stir for 24 hours at room temperature. Benzoquinone (20 mg, 0.19 mmol) and carbamazepine (88 mg, 0.37 mmol) were added to 0.5 mL acetonitrile and left to stir for 24 hours at room temperature. Benzoquinone (40 mg, 0.37 mmol) and carbamazepine (166 mg, 0.71 mmol) were added to 0.5 mL ethyl acetate and left to stir for 24 hours at room temperature. Benzoquinone (20 mg, 0.19 mmol) and carbamazepine (88 mg, 0.37 mmol) were added to 0.5 mL methyl ethyl ketone and left to stir for 24 hours at room temperature.

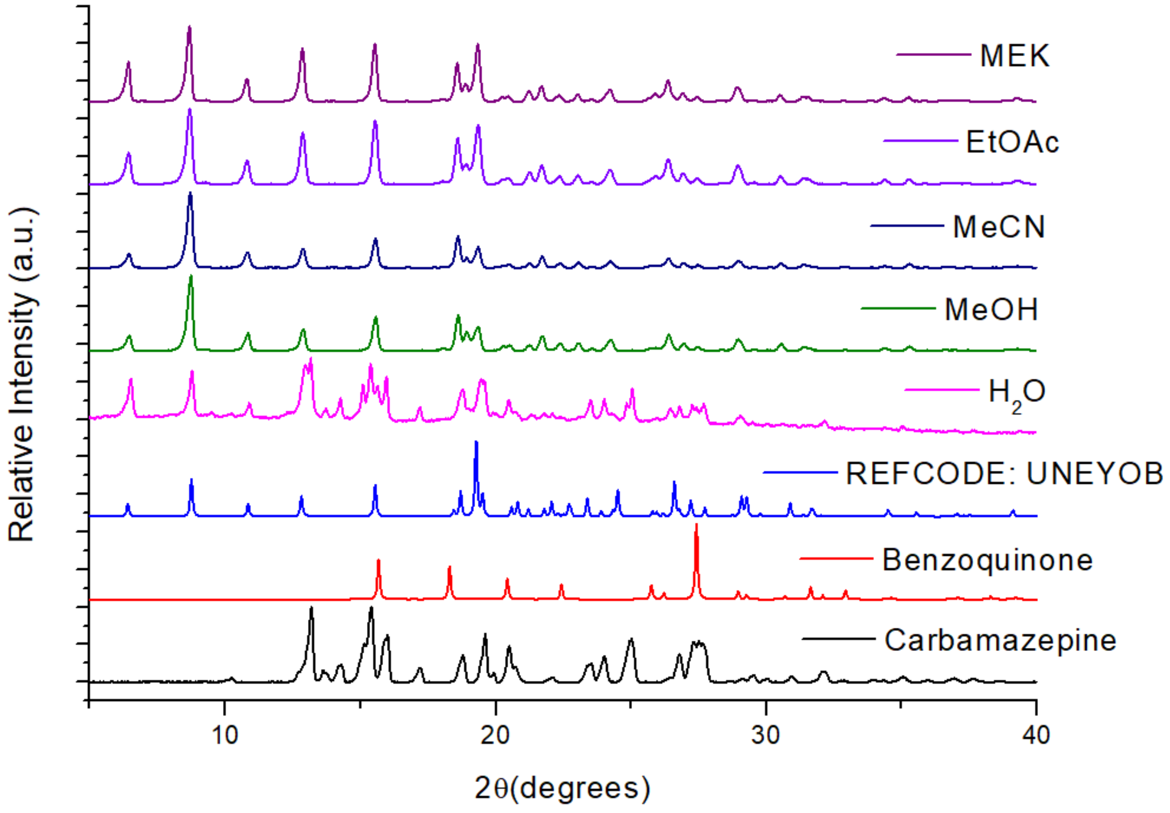

Figure S21*. PXRD patterns from slurries in (top) MEK, EtOAc, MeCN, MeOH, water, cocrystal 21 calculated PXRD and the two starting materials.*

Slurry 22: Terephthalaldehyde · Carbamazepine (1:2) [REFCODE: UNEYUH]

Terephthalaldehyde (40 mg, 0.29 mmol) and carbamazepine (136 mg, 0.60 mmol) were added to 0.5 mL deionized water and left to stir for 48 hours at room temperature. Terephthalaldehyde (45mg, 0.45 mmol) and carbamazepine (211 mg, 0.90 mmol) were added to 0.4 mL methanol and left to stir for 24 hours at room temperature. Terephthalaldehyde (50 mg, 0.37 mmol) and carbamazepine (166 mg, 0.71 mmol) were added to 0.5 mL acetonitrile and left to stir for 24 hours at room temperature. Terephthalaldehyde (50 mg, 0.37 mmol) and carbamazepine (166 mg, 0.71 mmol) were added to 0.5 mL ethyl acetate and left to stir for 24 hours at room temperature. Terephthalaldehyde (50 mg, 0.37 mmol) and carbamazepine (166 mg, 0.71 mmol) were added to 0.5 mL methyl ethyl ketone and left to stir for 24 hours at room temperature.

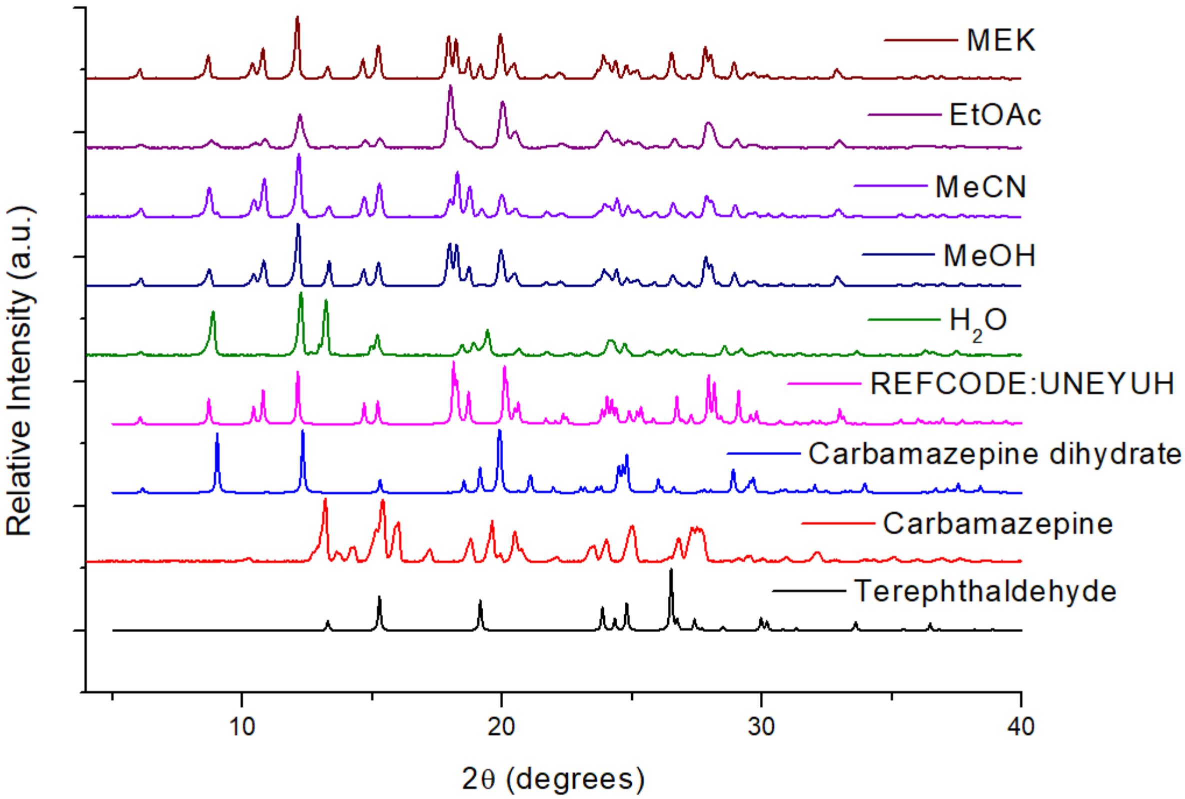

Figure S22. *PXRD patterns from slurries in (top) MEK, EtOAc, MeCN, MeOH, water, cocrystal 22 calculated PXRD and the two starting materials.*

Slurry 23: Saccharin · Carbamazepine (1:1) [REFCODE: UNEZAO]

Saccharin (50 mg, 0.27 mmol) and carbamazepine (65 mg, 0.28 mmol) were added to 0.5 mL deionized water and left to stir for 48 hours at room temperature. Saccharin (50 mg, 0.27 mmol) and carbamazepine (65 mg, 0.28 mmol) were added to methanol and left to stir for 24 hours at room temperature. Saccharin (50 mg, 0.27 mmol) and carbamazepine (65 mg, 0.28 mmol) were added to 0.5 mL acetonitrile and left to stir for 24 hours at room temperature. Saccharin (50 mg, 0.27 mmol) and carbamazepine (65 mg, 0.28 mmol) were added to 0.5 mL ethyl acetate and left to stir for 24 hours at room temperature. Saccharin (50 mg, 0.27 mmol) and carbamazepine (65 mg, 0.28 mmol) were added to 0.5 mL methyl ethyl ketone and left to stir for 24 hours at room temperature.

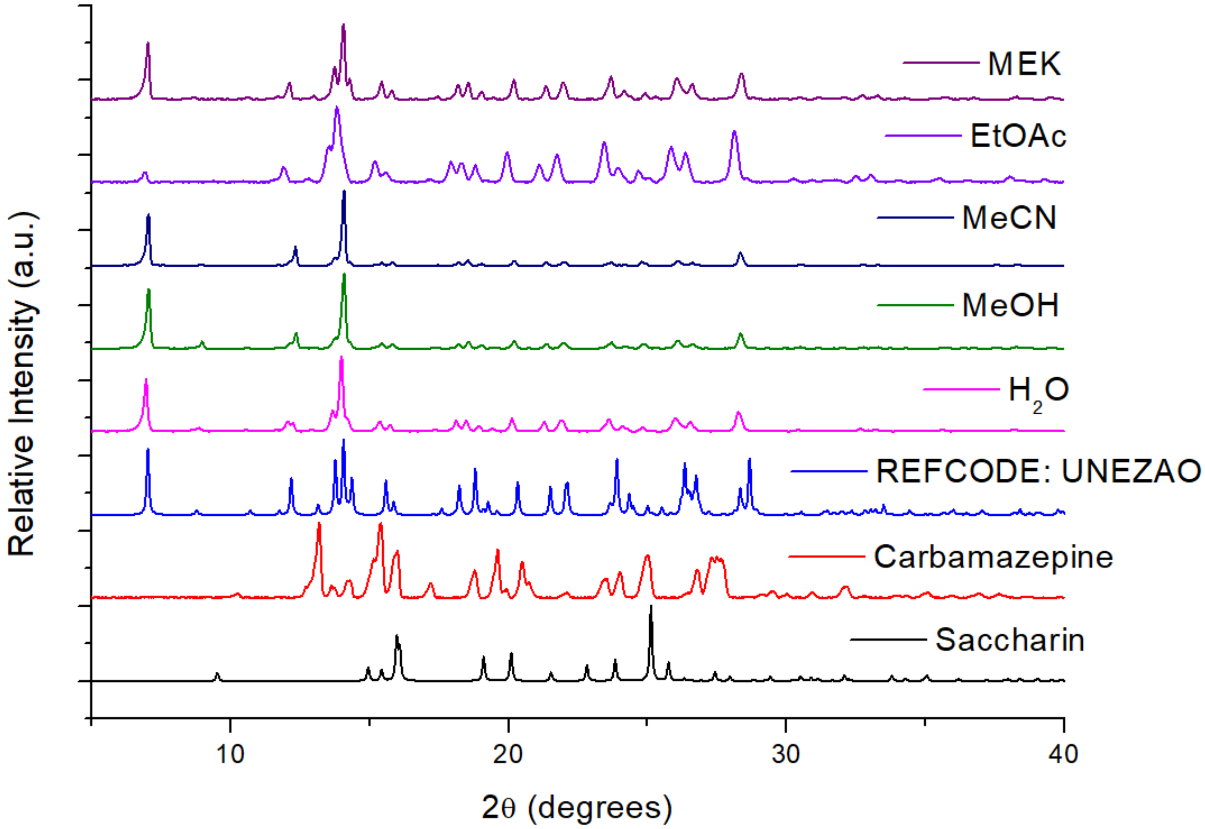

***Figure S23**. PXRD patterns from slurries in (top) MEK, EtOAc, MeCN, MeOH, water, cocrystal 23 calculated PXRD and the two starting materials.*

Slurry 24: Nicotinamide · Carbamazepine (1:1) [REFCODE: UNEZES]

Nicotinamide (110 mg, 0.9 mmol) and carbamazepine (212 mg, 0.9 mmol) were added to 0.5 mL deionized water and left to stir for 48 hours at room temperature. Nicotinamide (110 mg, 0.9 mmol) and carbamazepine (212 mg, 0.9 mmol) were added to methanol and left to stir for 24 hours at room temperature. Nicotinamide (52 mg, 0.42 mmol) and carbamazepine (100 mg, 0.42 mmol) were added to 0.5 mL acetonitrile and left to stir for 24 hours at room temperature. Nicotinamide (52 mg, 0.42 mmol) and carbamazepine (100 mg, 0.42 mmol) were added to 0.5 mL ethyl acetate and left to stir for 24 hours at room temperature. Nicotinamide (52 mg, 0.42 mmol) and carbamazepine (100 mg, 0.42 mmol) were added to 0.5 mL methyl ethyl ketone and left to stir for 24 hours at room temperature.

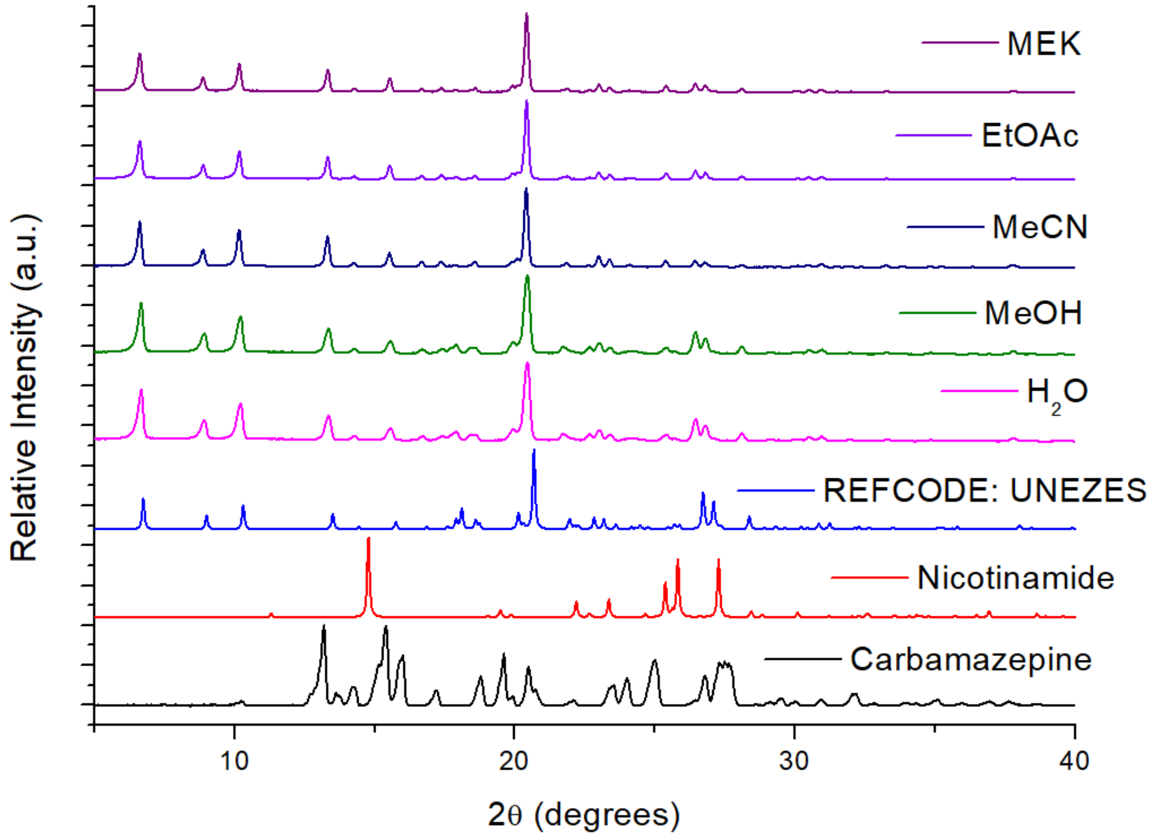

***Figure S24**. PXRD patterns from slurries in (top) MEK, EtOAc, MeCN, MeOH, water, cocrystal 24 calculated PXRD and the two starting materials.*

Slurry 25: Aspirin · Carbamazepine (1:1) [REFCODE: TAZROA]

Aspirin (50 mg, 0.28 mmol) and carbamazepine (66 mg, 0.28 mmol) were added to 0.5 mL deionized water and left to stir for 48 hours at room temperature. Aspirin (164 mg, 0.91 mmol) and carbamazepine (215 mg, 0.91 mmol) were added to 1 mL methanol and left to stir for 24 hours at room temperature. Aspirin (50 mg, 0.28 mmol) and carbamazepine (66 mg, 0.28 mmol) were added to 0.5 mL acetonitrile and left to stir for 24 hours at room temperature. Aspirin (50 mg, 0.28 mmol) and carbamazepine (66 mg, 0.28 mmol) were added to 0.5 mL ethyl acetate and left to stir for 24 hours at room temperature. Aspirin (75 mg, 0.42 mmol) and carbamazepine (98 mg, 0.42 mmol) were added to 0.5 mL methyl ethyl ketone and left to stir for 24 hours at room temperature.

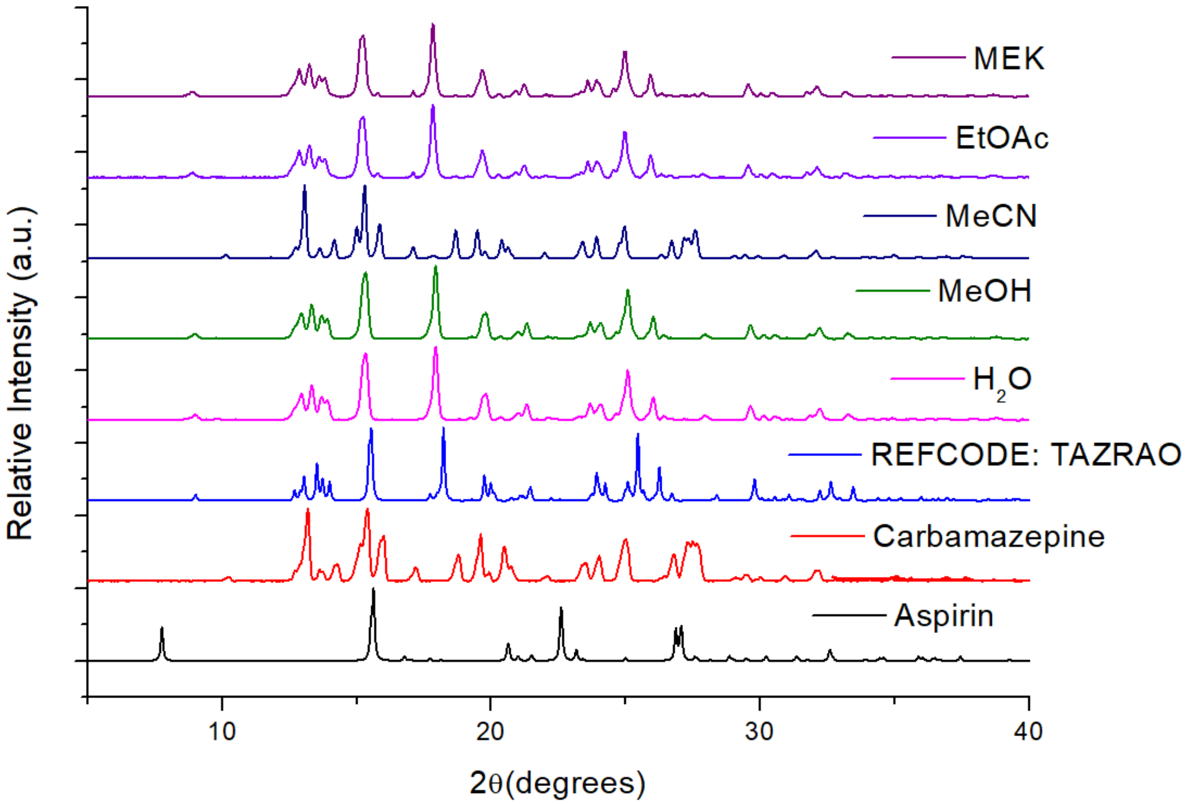

Figure S25. *PXRD patterns from slurries in (top) MEK, EtOAc, MeCN, MeOH, water, cocrystal 25 calculated PXRD and the two starting materials.*
